# Exploring the mechanism of anti-chronic heart failure effect of qiweiqiangxin І granules based on metabolomics

**DOI:** 10.3389/fphar.2023.1111007

**Published:** 2023-02-13

**Authors:** Wanru Zhong, Yihua Li, Haixiang Zhong, Yuanyuan Cheng, Qi Chen, Xinjun Zhao, Zhongqiu Liu, Rong Li, Rong Zhang

**Affiliations:** ^1^ Guangdong Provincial Key Laboratory of Translational Cancer Research of Chinese Medicines, Joint International Research Laboratory of Translational Cancer Research of Chinese Medicines, School of Pharmaceutical Sciences, International Institute for Translational Chinese Medicine, Guangzhou University of Chinese Medicine, Guangzhou, China; ^2^ The first clinical medical college, Guangzhou University of Chinese Medicine, Guangzhou, China; ^3^ Department of Internal Medicine-Cardiovascular, The First Affiliated Hospital of Guangzhou University of Chinese Medicine, Guangzhou, Guangdong, China

**Keywords:** chronic heart failure, randomized controlled clinical trials, pharmacodynamics, metabolomics, pathway analysis, efficacy

## Abstract

**Background:** Qiweiqiangxin І granules (QWQX І) is a traditional Chinese medicine preparation based on the basic theory of traditional Chinese medicine, which produces a good curative effect in treating chronic heart failure (CHF). However, its pharmacological effect and potential mechanism for CHF remain unknown.

**Aim of the study:** The purpose of this study is to clarify the efficacy of QWQX І and its possible mechanisms.

**Materials and methods:** A total of 66 patients with CHF were recruited and randomly assigned to the control or QWQX І groups. The primary endpoint was the effect of left ventricular ejection fraction (LVEF) after 4 weeks of treatment. The LAD artery of rats was occluded to establish the model of CHF. Echocardiography, HE and Masson staining were performed to evaluate the pharmacological effect of QWQX І against CHF. Ultra-performance liquid chromatography-quadrupole time-of-flight mass spectrometry (UPLC-QTOF/MS) untargeted metabolomics was to screen endogenous metabolites in rat plasma and heart and elucidate the mechanism of QWQX І against CHF. Results: In the clinical study, a total of 63 heart failure patients completed the 4-week follow-up, including 32 in the control group and 31 in QWQX І group. After 4 weeks of treatment, LVEF was significantly improved in QWQX І group compared with the control group. In addition, the patients in QWQX І group had better quality of life than the control group. In animal studies, QWQX І significantly improved cardiac function, decreased B-type natriuretic peptide (BNP) levels, reduced inflammatory cell infiltration, and inhibited collagen fibril rate. Untargeted metabolomic analysis revealed that 23 and 34 differential metabolites were screened in the plasma and heart of chronic heart failure rats, respectively. 17 and 32 differential metabolites appeared in plasma and heart tissue after QWQX І treatment, which were enriched to taurine and hypotaurine metabolism, glycerophospholipid metabolism and linolenic acid metabolism by KEGG analysis. LysoPC (16:1 (9Z)) is a common differential metabolite in plasma and heart, which is produced by lipoprotein-associated phospholipase A2 (Lp-PLA2), hydrolyzes oxidized linoleic acid to produce pro-inflammatory substances. QWQX І regulates the level of LysoPC (16:1 (9Z)) and Lp-PLA2 to normal.

**Conclusion:** QWQX І combined with western medicine can improve the cardiac function of patients with CHF. QWQX І can effectively improve the cardiac function of LAD-induced CHF rats through regulating glycerophospholipid metabolism and linolenic acid metabolism-mediated inflammatory response. Thus, QWQX I might provide a potential strategy for CHF therapy.

## 1 Introduction

Chronic heart failure (CHF) has been recognized as a major clinical and public health problem (Ambrosy et al., 2014). Globally, an estimated 64.3 million people suffer from CHF and more than 1 million are hospitalized per year in the United States and Europe (Groenewegen et al., 2020). Coronary heart disease and hypertension are the most important causes for CHF(Ziaeian and Fonarow, 2016). CHF is a complex clinical syndrome characterized by structural changes or functional abnormalities of the heart (Ponikowski et al., 2016), which is a severe manifestation or end-stage of various heart diseases (Metra and Teerlink, 2017). Current treatment focuses on inhibiting abnormal neuroendocrine activation (Sayer and Bhat, 2014), including inhibitors of angiotensin-converting enzyme (ACEI), inhibitors of angiotensin II receptors (ARBs), inhibitors of angiotensin receptor enkephalinase (ARINs), beta-blockers, and antagonists of aldosterone receptors (McDonagh et al., 2021). The latest research shows that sodium-glucose cotransporter 2 (SGLT2) inhibitors reduced the risk of hospitalizations for heart failure (Nassif et al., 2021). However, CHF patients after the current treatment still have poor prognosis and low 5-year survival rate (Rosano and Vitale, 2018). Therefore, it is still urgent to find a new strategy for CHF therapy at this stage (Zhuang et al., 2021).

Traditional Chinese medicine (TCM) has provided ideas for the treatment of various diseases over the years (Wen et al., 2018). TCM has accumulated rich clinical experience in the treatment of HF, and has unique curative effects in stabilizing heart failure, improving cardiac function and quality of life (Wang et al., 2017). In TCM, qi deficiency, static blood, and water retention are the basic pathogenesis of HF. Professor Rong Li proposed the theory “Golden Triangle of Traditional Chinese Medicine” for CHF therapy based on TCM theory and clinical experiences (Chen et al., 2021). The basic prescription for HF treatment called Qiweiqiangxin І (QWQX І) was created according to the theory. QWQX І consists of seven traditional Chinese medicines including Renshen (*Panax ginseng* C. A. Meyer.), Huangqi (*Astragalus membranaceus* (Fisch.) Bge. var. *mongholicus* (Bge.) Hsiao), Danshen (*SaZwia miltiorrhiza* Bge.), Yimucao (*Leonurus japonicus* Houtt.), Tinglizi (*Descurainia sophia* (L.) Webb, e x Prantl.), Maodongqing (*Ilex pubescens*) and Guizhi (*Cinnamomum cassia* Preslf). Previous studies showed that the single Chinese herbal in QWQX І could improve cardiac function, reducing myocardial fibrosis and maintaining circulatory system homeostasis in CHF (Bernatoniene et al., 2014; Zhang et al., 2021; Zheng et al., 2022). Active ingredients in QWQX І, such as ginsenoside Re and cinnamon sticks, which can play a role in the treatment of heart damage (Wang et al., 2019; Peng et al., 2021). QWQX І has a complex chemical composition and multiple targets. Whether and how it reverses CHF cardiac remodeling after myocardial infarction (MI) in rats remains unknown. Therefore, it is necessary to establish a good matching relationship between its pharmacodynamic material basis, pharmacodynamics and mechanism of action, hindering the further application of QWQX І.

Metabolomics is an emerging discipline in recent years (Yu et al., 2017), which is helpful to reveal the regulatory mechanism of TCM. CHF is associated with a complex set of pathologies, including profound changes in cardiometabolism (Brankovic et al., 2018). Untargeted metabolomics is the comprehensive and systematic detection for all metabolites in the body, which is widely used to study the mechanism of disease occurrence and development, such as HF (Liu et al., 2018). Currently, UPLC-QTOF/MS is currently the most commonly used analytical method for metabolomics due to its wide range of metabolites, high throughput and high sensitivity (Cui et al., 2018). Several products of multiple cardiometabolic pathways, including metabolites related to inflammation and energy metabolism, are important intermediates in the pathogenesis of heart failure (Bacmeister et al., 2019). Thus, the application of metabolomics analyzed by UHPLC-QTOF/MS is contributed to explore the mechanism of QWQX І against CHF.

**FIGURE 1:**
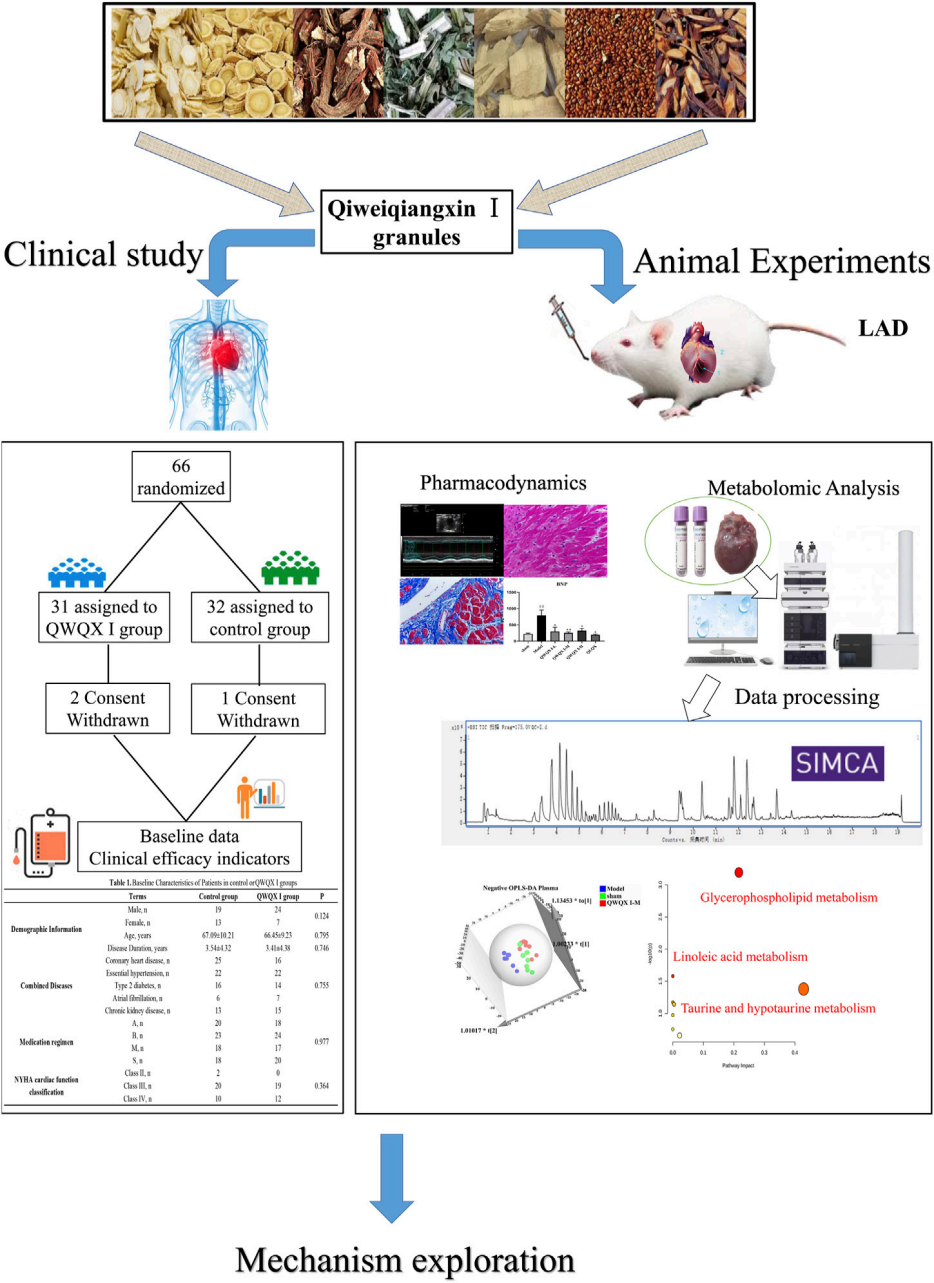
Research framework of this study.

In the present study, we firstly investigated the clinical effect of QWQX І treatment in CHF patients, and then evaluated the pharmacological effect of QWQX І in LAD-induced CHF rats. In addition, plasma and cardiac tissue metabolomics studies were performed using UPLC-QTOF/MS to explore the potential differential metabolites and key signaling pathways in CHF rats after QWQX І treatment. This study will provide a better understanding of the mechanism on QWQX І against CHF and alternative strategies for CHF prevention and treatment. The research process of this study is shown in Figure 1.

## 2 Materials and methods

### 2.1 Reagents and chemicals

Methanol, acetonitrile, formic acid and tamoxifen for chromatography grade were purchased from Merck (Darmstadt, Germany). QWQX І granules were obtained from China Resources Sanjiu Medical & Pharmaceutical CO., LTD (Shenzhen, China) (batch number 2205001H), UHPLC-QTOF/MS analysis of QWQX І granules was performed (Supplementary Figure S1; Supplementary Table S1). Qiliqiangxin capsule (QLQX) is purchased from Shijiazhuang Yiling Pharmaceutical Co., Ltd (batch number A2111025). Ultrapure water was produced by a Milli-Q system (Millipore, MA, United States).

### 2.2 Clinical studies

#### 2.2.1 Study design

The primary objective of the clinical study was to evaluate the therapeutic effect of QWQX І in patients with CHF. This study is a single-center, randomized, controlled, prospective clinical trial conducted in the First Affiliated Hospital of Guangzhou University of Chinese Medicine (NO.JY [2021]093).

#### 2.2.2 Eligibility criteria

##### 2.2.2.1 Inclusion criteria

I. Meet the diagnostic criteria of heart failure.
II. Age 40–80 years, gender not limited.
III. NYHA cardiac function class II-IV.
IV. Participate voluntarily, understand and sign the statement of consent.

##### 2.2.2.2 Exclusion criteria

I. Heart failure caused by valvular disease, precordial disease, or pericardial disease.
II. Patients with tumors, severe endocrine system disease, psychiatric disease, severe primary disease of the hematopoietic system, uncontrollable malignant arrhythmias, second-degree type II or greater sinus or atrioventricular block without pacemaker protection, progressive exacerbation of acute coronary syndrome, uncontrolled hypertensive crisis, hypokalemia.
III. Patients planning to receive coronary revascularization therapy or cardiac resynchronization therapy within 1 month.
IV. Women who are pregnant or planning to become pregnant and women who are breastfeeding for a short period of time.
V. Allergic patients or those with known allergy to therapeutic drugs.
VI. Those who are undergoing other drug trials.

#### 2.2.3 Study protocol

Participants who met all inclusion criteria and any of the exclusion criteria were randomly assigned to control group or QWQX І group. The control group received standardized anti-heart failure drug therapy (ACEI/ARB/ARNI, beta-blockers, antagonists of aldosterone receptors and SGLT2) according to the “2018 Chinese Guidelines for the Diagnosis and Treatment of Heart Failure”, and patients in the QWQX І group received QWQX I and the standardized anti-heart failure drug therapy. Baseline data was collected from patients on the day of enrollment, including gender, age, duration of HF, comorbid diseases, and anti-HF drug regimen. Primary and secondary endpoints for this study were collected at enrollment and after 4 weeks of follow-up, respectively.

#### 2.2.4 Endpoints

The primary endpoint of this clinical study was left ventricular ejection fraction (LVEF) as measured by echocardiography, and secondary endpoints included left ventricular end-diastolic internal diameter (LVEDD), left ventricular end-systolic internal diameter (LVESD), plasma N-terminal pro-B type natriuretic peptide (NT-proBNP) levels, and the Minnesota Life with Heart Failure Questionnaire (MLHFQ) score.

### 2.3 Pharmacodynamic analysis

#### 2.3.1 Animal models and research design

Male Sparaque-Dawley rats (body weight 220–250 g) from the Animal Experiment Center of Southern Medical University. Experimental procedures were approved by the Animal Ethical Use Committee of Guangzhou University of Traditional Chinese Medicine (No. IITCM-20211213).

After anesthesia, the rats were subjected to the ventilator and the pericardium was opened. The left anterior descending coronary artery (LAD) was ligated with a 6–0 polypropylene suture. The myocardium below the ligation point turns red to white, which indicated that a model of myocardial ischemia (MI) was successfully established. A similar surgery procedure was performed without LAD ligation in Sham group.

The rats with MI were randomized into 6 groups after 4 weeks: sham group, model group, QLQX group (600 mg/kg), low-dose QWQX І group (QWQX І-L, 850 mg/kg, equivalent to 0.5 time of the clinical dose), medium-dose QWQX І group (QWQX І-M, 1700 mg/kg, a dose equivalent to the clinical dose) and a high-dose QWQX І group (QWQX І-H, 3400 mg/kg, which corresponds to twice of the clinical dose). The model group and the sham group were given the same dose of deionized water. Low, medium and high dose groups received QWQX І. Gastric suspension was administered to the QLQX group. The above 6 groups were treated with continuous medication or distilled water for 4 weeks.

#### 2.3.2 Echocardiographic assessment of cardiac function

Cardiac geometry and function were assessed by echocardiographic analysis using a 30 MHz high-resolution imaging system radiofrequency scan head (VisualSonics Vevo770, VisualSonics, Toronto, Canada).

#### 2.3.3 Plasma and cardiac tissue sample collection

Plasma and cardiac tissue were collected after completion of the 8-week echocardiographic study. All animals were anesthetized by intraperitoneal injection of 40 mg/kg 2% sodium pentobarbital. Blood was collected from the abdominal aorta and plasma was prepared as follows: the blood was placed in purple capped tubes and left at room temperature for 30 min. The tubes were placed in a refrigerated centrifuge (4°C) and spun at 4000 rpm for 15 min. Transfer the supernatant to cryovials and snap freeze in liquid nitrogen. Expose the heart and inject KCl (0.1 mol/L) through the apex of the left ventricle until diastole stops. Hearts were immediately removed and washed in ice-cold saline and snap frozen in liquid nitrogen. Plasma and cardiac tissue samples were stored in a −80°C freezer until metabolomic analysis.

#### 2.3.4 ELISA kit for the detection of BNP in plasma

Rat peripheral plasma BNP was determined using ELISA kit (Quanzhou Ruixin Biology, Hangzhou, China). Measure the absorbance at OD of 450 nm using a Thermofisher microplate reader according to instructions.

#### 2.3.5 Histological examination

##### 2.3.5.1 Hematoxylin-eosin staining

After paraffin-embedding the sections, the sections were alternately treated with xylene, 100%, 95%, 80%, and 70% ethanol and distilled water for 5 min, respectively. Then, sections were stained with hematoxylin for 5 min and blued in water. After differentiation with 0.5% HCl/EtOH solution for 5 s, sections were stained with eosin for 5 min. After that, the sections were observed and photographed under a light microscope.

##### 2.3.5.2 Masson staining

The cardiac tissue of each group was harvested, dehydrated with graded ethanol, completely immersed in xylene, embedded in paraffin, routinely sectioned, after melting the wax on the paraffin sections, the sections were soaked in potassium dichromate overnight, washed in tap water, incubated in iron hematoxylin for 3 min, and differentiated with hydrochloric acid ethanol solution. Then, the slices were dipped with ponceau red acid fuchsin (5–10 min), and washed with tap water. After that, the slices were dipped with phosphomolybdic acid aqueous solution for 1–3 min and stained directly with aniline blue dye solution (3–6 min). Finally, the slices were differentiated with 1% glacial acetic acid, dehydrated with absolute ethanol, a sealed with neutral gum and examined for myocardial fibrosis by microscopy. The fibrotic area was analyzed using image analysis software (Image Pro Plus 6.0, Media Cybernetics, United States) and the ratio of the area of the blue area (fibrotic area) to the area of the red area (normal myocardium) was calculated as collagen volume fraction. Myocardial collagen content was expressed as collagen volume fraction (CVF).

### 2.4 Metabolomic analysis

#### 2.4.1 Metabolite extraction

##### 2.4.1.1 Plasma sample preparation

After thawing on ice, 50 µl plasma was mixed with 150 µl MeOH, vortexed for 3 min, kept overnight at −20°C, and then centrifuged at 14,000 rpm for 15 min at 4°C. Next, the supernatant was collected and dried under vacuum in 100 µl MeOH:H2O (1:1, v:v) at room temperature through a SpeedVac concentrator (Savant™ SPD1010, Thermo Scientific, Shanghai, China), internal standard Reconstituted (500 nM tamoxifen and d_4_-CA) and centrifuged at 14,000 rpm for 30 min at 4°C prior to analysis.

##### 2.4.1.2 Cardiac tissue sample preparation

50 mg of cardiac tissue were mixed with 1 ml of MeOH and four small steel beads for homogenization by using an automated homogenizer (Tissuelyser-24, Jingxin Industrial Development Co., Ltd., Shanghai, China). Tissues were homogenized for 3 cycles and stored at -20°C for 1 h, then centrifuged at 14,000 rpm for 15 min at 4°C. Next, the supernatant was collected and dried *in vacuo* at room temperature through a SpeedVac concentrator (Savant™ SPD1010, Thermo Scientific, Shanghai, China), and mixed in 100 µl MeOH:H_2_O (1:1, v:v) and internal standard (500 nM tamoxifen and d_4_-CA) and centrifuged at 14,000 rpm for 30 min at 4 °C prior to analysis.

##### 2.4.1.3 Quality control samples preparation

An equal amount of plasma or cardiac homogenate was taken from each sample, mixed well, and processed in the same way as the sample processing method.

#### 2.4.2 UHPLC-QTOF/MS analysis

UHPLC-QTOF/MS analysis was performed using a 1290 ultra-high performance liquid chromatography system (Agilent, California, United States) and a 6540 Q-TOF mass spectrometer (Agilent, California, United States), operated in positive and negative ion modes. MS parameter settings are shown in Supplementary Table S2. Analysis were separated on a Waters BEH C18 column (2.1 × 150 mm, 1.7 μm, Milford, MA, United States) at a constant flow rate of 0.4 ml/min at a column temperature of 50°C. LC conditions were as follows: 0.1% formic acid in deionized water (solvent A) and 0.1% formic acid in acetonitrile (solvent B); gradient elution method is shown in Supplementary Table S3.

#### 2.4.3 Metabolomics data processing

LC-MS data was acquired using Agilent MassHunter Acquisition software, and the data was batch processed using ProFinder 10.0 and Mass Profiler Professional software to extract characteristic information for characteristic ion peaks. Peak areas were log-transformed and finally normalized using the total peak area normalization method. A table containing ion identities, retention times, and normalized peak areas was imported into SIMCA-P for data analysis. The principle component analysis (PCA) and orthogonal projection to latent structures-discriminant analysis (OPLS-DA) model has been overfit validated and is only used if it is not overfit. Differential metabolites were screened according to the criteria of Variable Importance in Projection (VIP) > 1 and *p* < 0.05. MetaboAnalyst 5.0 was used for metabolic pathway analysis.

### 2.5 Statistical analysis

The results of the measurement data were expressed as the Mean ± SEM, and were analyzed using SPSS software (version 20.0, IBM, Chicago, Illinois, United States). If normality and homogeneity of variances were met, the differences among the groups were compared using one-way analysis of variance (0ne-way ANOVA); Bonferroni test was used for pairwise comparisons; if the variances were not homogeneous, Dunnett’s T3 test was used. If the normal distribution was not satisfied, non-parametric analysis was used to test the difference. Count data were analyzed by chi-square test or Fisher’s exact probability method. *p* < 0.05 means the difference is statistically significant.

## 3 Results

### 3.1 QWQX І combined with western medicine can improve CHF patients’ cardiac function

A total of 66 patients with heart failure were included in this study, of which 3 (1 in the control group and 2 in the QWQX І group) were dislodged due to inaccessibility, resulting in 32 in the control group and 31 in the QWQX І group. Baseline data for the two groups are presented in Table 1. After statistical analysis, there were no statistically significant differences between the two groups at baseline in terms of age, gender, disease duration, comorbidities, and medication regimen (*p* > 0.05). As shown in Table 2, in the control group, the mean LVEF of patients after 4 weeks of treatment was 36.19 ± 10.39%, with no statistically significant differences before and after treatment (*p* > 0.05). In contrast, the mean LVEF in the QWQXІ group after treatment was 42.10 ± 12.38%, which was significantly higher than before treatment (*p* < 0.01). There was also a statistically significant difference in LVEF between the two groups (-1.19 ± 8.12% vs. 4.12 ± 9.15%, *p* < 0.01). LVESD, plasma NT-proBNP levels and MLHFQ scores decreased statistically after treatment in the QWQX І group compared with before treatment (*p* < 0.05), and MLHFQ scores decreased before and after treatment in both groups (*p* < 0.05); however, there was no statistically significant difference in LVEDD, LVESD and plasma NT-proBNP before and after treatment between the two groups (*p* > 0.05).

**TABLE 1:** Baseline Characteristics of Patients in control or QWQX І groups.

	Terms	Control group	QWQX І group	*P*
**Demographic Information**	Male, n	19	24	0.124
	Female, n	13	7	
	Age, years	67.09 ± 10.21	66.45 ± 9.23	0.795
	Disease Duration, years	3.54 ± 4.32	3.41 ± 4.38	0.746
**Combined Diseases**	Coronary heart disease, n	25	16	0.755
	Essential hypertension, n	22	22	
	Type 2 diabetes, n	16	14	
	Atrial fibrillation, n	6	7	
	Chronic kidney disease, n	13	15	
**Medication regimen**	A, n	20	18	0.977
	B, n	23	24	
	M, n	18	17	
	S, n	18	20	
**NYHA cardiac function classification**	Class II, n	2	0	0.364
	Class III, n	20	19	
	Class IV, n	10	12	

A = angiotensin-converting enzyme inhibitors/angiotensin receptor blockers/angiotensin receptor-neprilysin inhibitors, B = adrenergic beta-blockers, M = mineralocorticoid receptor antagonists, S = sodium-glucose cotransporter 2 inhibitors.

**TABLE 2:** Change in endpoints from baseline to after 4 weeks of follow-up.

Endpoints	Groups	Baseline	4-week	*P*
LVEF	Control group	37.38 ± 12.44	36.19 ± 10.39	0.415
	QWQX І group	37.97 ± 11.95	42.10 ± 12.38	0.018
	P	0.848	*p* = 0.044	
LVEDD	Control group	61.66 ± 9.73	61.84 ± 9.11	0.887
	QWQX І group	60.71 ± 9.68	60.55 ± 9.05	0.880
	P	0.700	0.573	
LVESD	Control group	50.16 ± 9.44	50.50 ± 9.80	0.741
	QWQX І group	49.65 ± 10.56	47.61 ± 10.13	0.047
	P	0.840	0.255	
NT-proBNP levels	Control group	2221.15 (822.80, 7085.94)	1230.36 (452.80, 2590.75)	0.010
	QWQX І group	1331.00 (827.05, 2609.50)	927.80 (382.60, 1910.00)	0.000
	P	0.111	0.402	
MLHFQ score	Control group	60.59 ± 7.04	43.94 ± 7.94	0.000
	QWQX І group	61.65 ± 5.33	39.26 ± 5.65	0.000
	P	0.506	0.009	

### 3.2 QWQX І treatment improved cardiac function and decreased BNP in CHF rats

**FIGURE 2:**
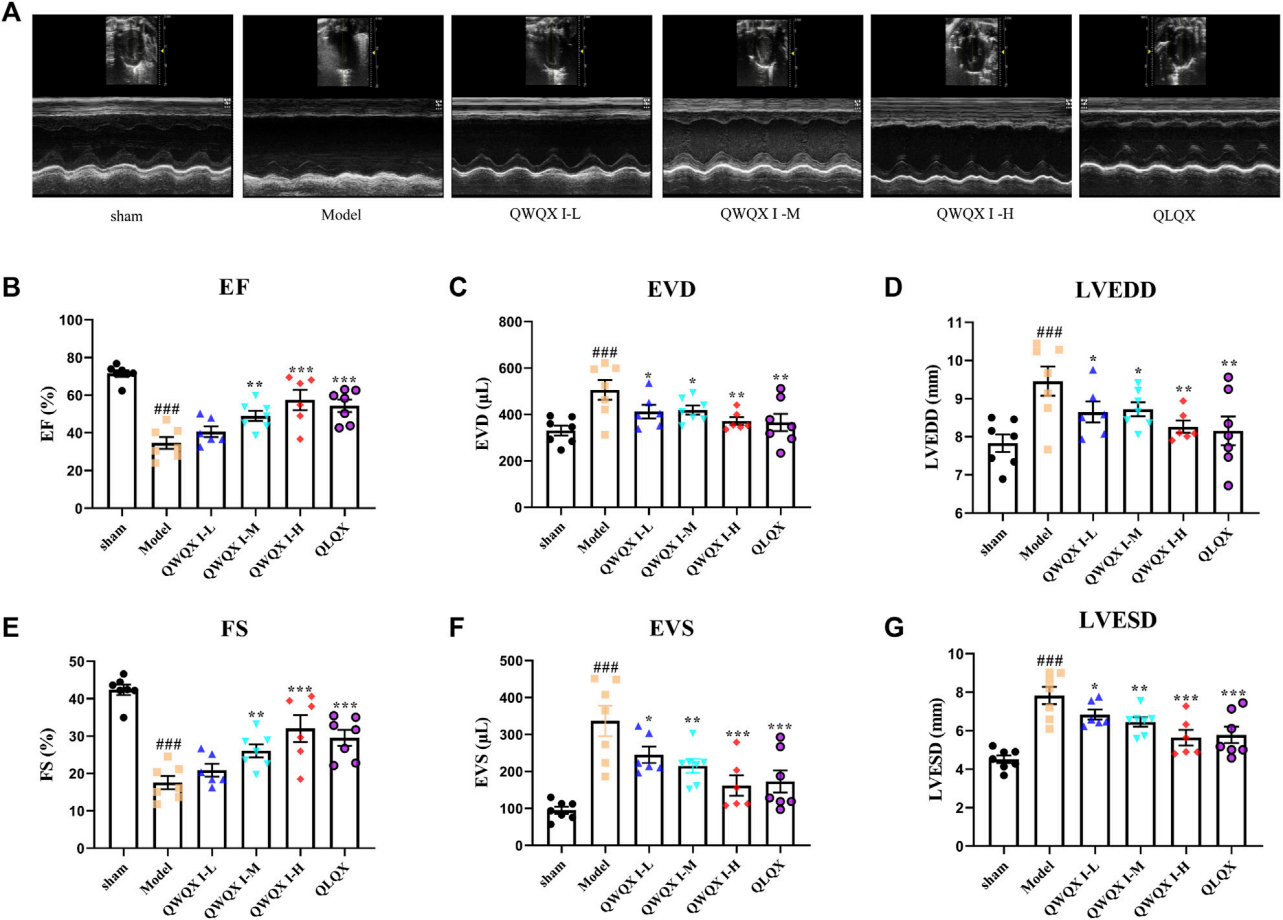
QWQX І treatment improved cardiac function in CHF rats. **(A)** Echocardiography images of rats in different groups. **(B–G)** Effect of QWQX І on LAD-induced echocardiographic parameters. Data are expressed as the Mean ± SEM. ^##^ *p* < 0.01, ^###^ *p* < 0.001, the Model group vs. the sham group; *p < 0.05, **p < 0.01, ***p < 0.001, drug treatment group vs. the Model group.

**FIGURE 3:**
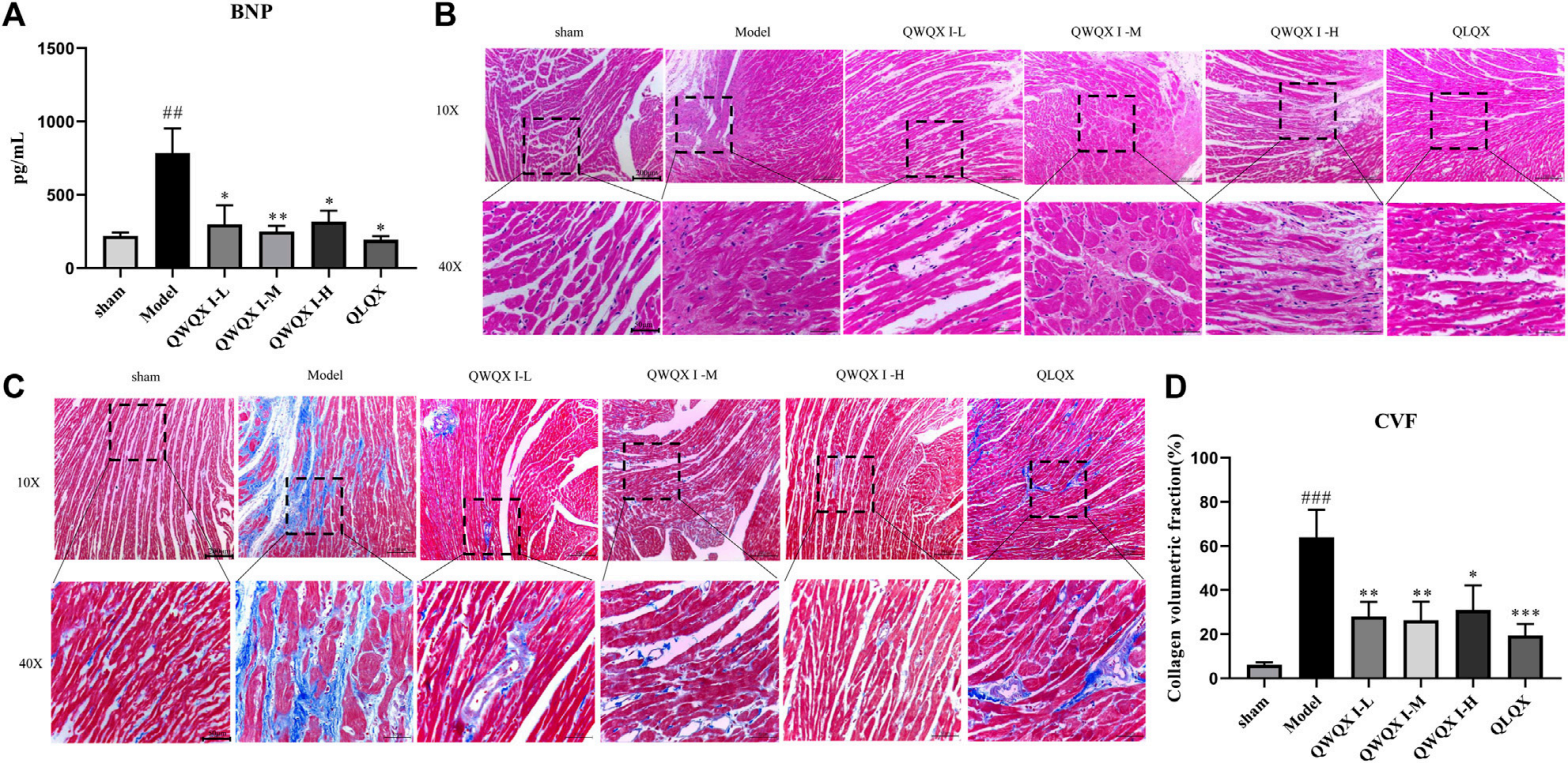
Effect of QWQX І on histopathological changes in CHF rats. **(A)**Plasma level of BNP. **(B)** Images of HE staining. **(C)** Images of Masson staining. **(D)** Quantitative analysis of CVF. Data are expressed as the Mean ± SEM. ^###^ *p* < 0.001, the Model group vs. the sham group; *p < 0.05, **p < 0.01, ***p < 0.001, drug treatment group vs. the Model group.

As shown in Figures 2A–G, after 4 weeks of drug intervention, echocardiography was performed on the rats in each group. The echocardiography results showed that compared with the sham group, the EF and FS of the Model group decreased (*p* < 0.001) after LAD induction. LVEDD, LVESD, left ventricular end-systolic volume (EVS), and left ventricular end-diastolic volume (EVD) were all significantly decreased (*p* < 0.05). QWQX І treatment significantly increased the EF from 34.66% to 36.77%, 49.01%, 54.92%, and FS from 17.55% to 20.90%, 25.91%, 32.05% in a dose-dependent manner, which at QWQX І-M group was equivalent to the effect of QLQX treatment. In addition, LVEDD, LVESD, EVS and EVD were significantly reduced (*p* < 0.05). The plasma BNP levels of rats were detected, and the Model was significantly higher than the sham group in Figure 3A (*p* < 0.01). QWQW I and QLQX decreased the level of BNP (*p* < 0.05); and the BNP in the QWQX І-M group decreased significantly from 785.65 pg/ml to 248.98 pg/ml (*p* < 0.05). decreased (*p* < 0.01). The above results suggest that QWQX І can protect rats against LAD-induced CHF.

### 3.3 QWQX І treatment improved the pathological changes and cardiac fibrosis in CHF rats

HE staining in Figure 3B showed that compared with the sham group, there was a widely thinned infarct area in the left ventricular wall in the Model group. These pathological changes were relatively improved in the QLQX and QWQX І groups. Specifically, after QLQX and QWQX І treatment, the morphology of muscle fibers in the infarct border area was basically regular, and the number of necrotic muscle cells was reduced. As shown in Figure 3C, the normal muscle tissue of the sham group had only a small amount of collagen. However, a large amount of blue collagen was occupied in the infarcted area and interstitial area of muscle cells in the Model group. Based on Masson’s trichrome staining, CVF is the recommended index for evaluating left ventricular fibrosis. As shown in Figure 3D, compared with the sham group, the CVF of the Model group was significantly increased (*p* < 0.001), however, QLQX and QWQX І treatment partially reduced the collagen (*p* < 0.05).

### 3.4 QWQX І modulated the metabolome of plasma in CHF rats

Based on the clinical study and pharmacodynamic results, the sham group, Model group and QWQX І -M group were selected for UHPLC-QTOF/MS analysis.

**FIGURE 4:**
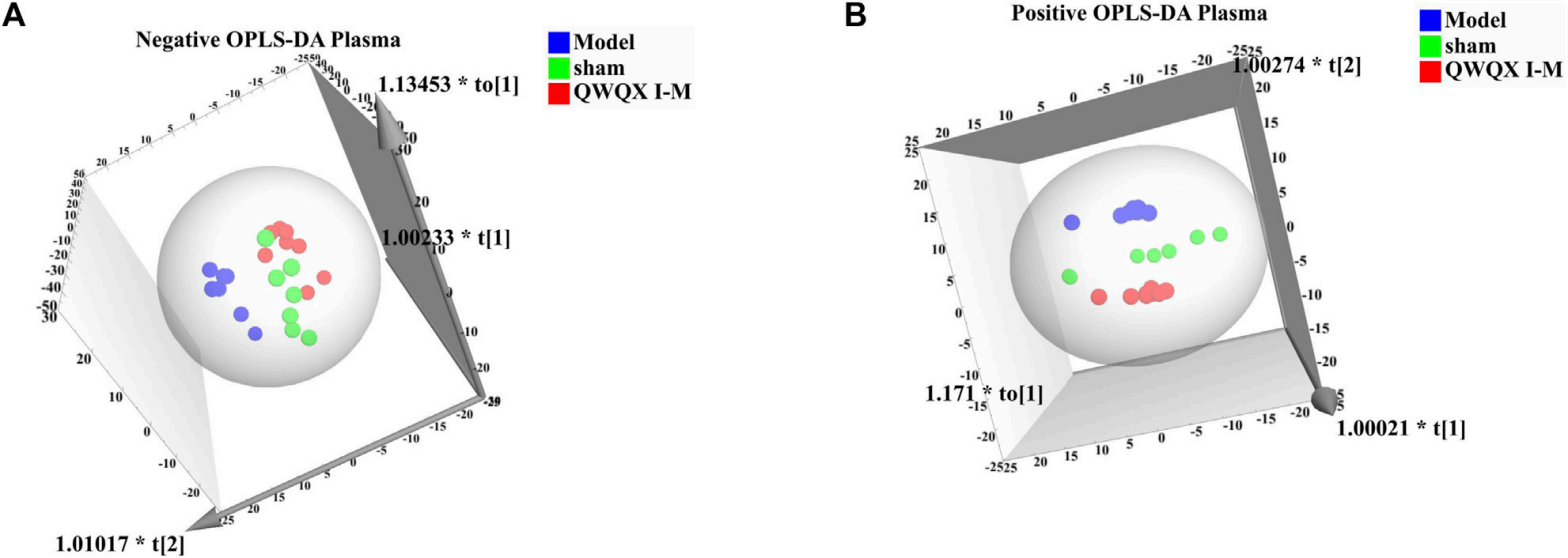
Plasma metabolomics OPLS-DA scores for each group of rats. **(A)** Negative mode of various rats. **(B)** Positive mode of various rats.

**FIGURE 5:**
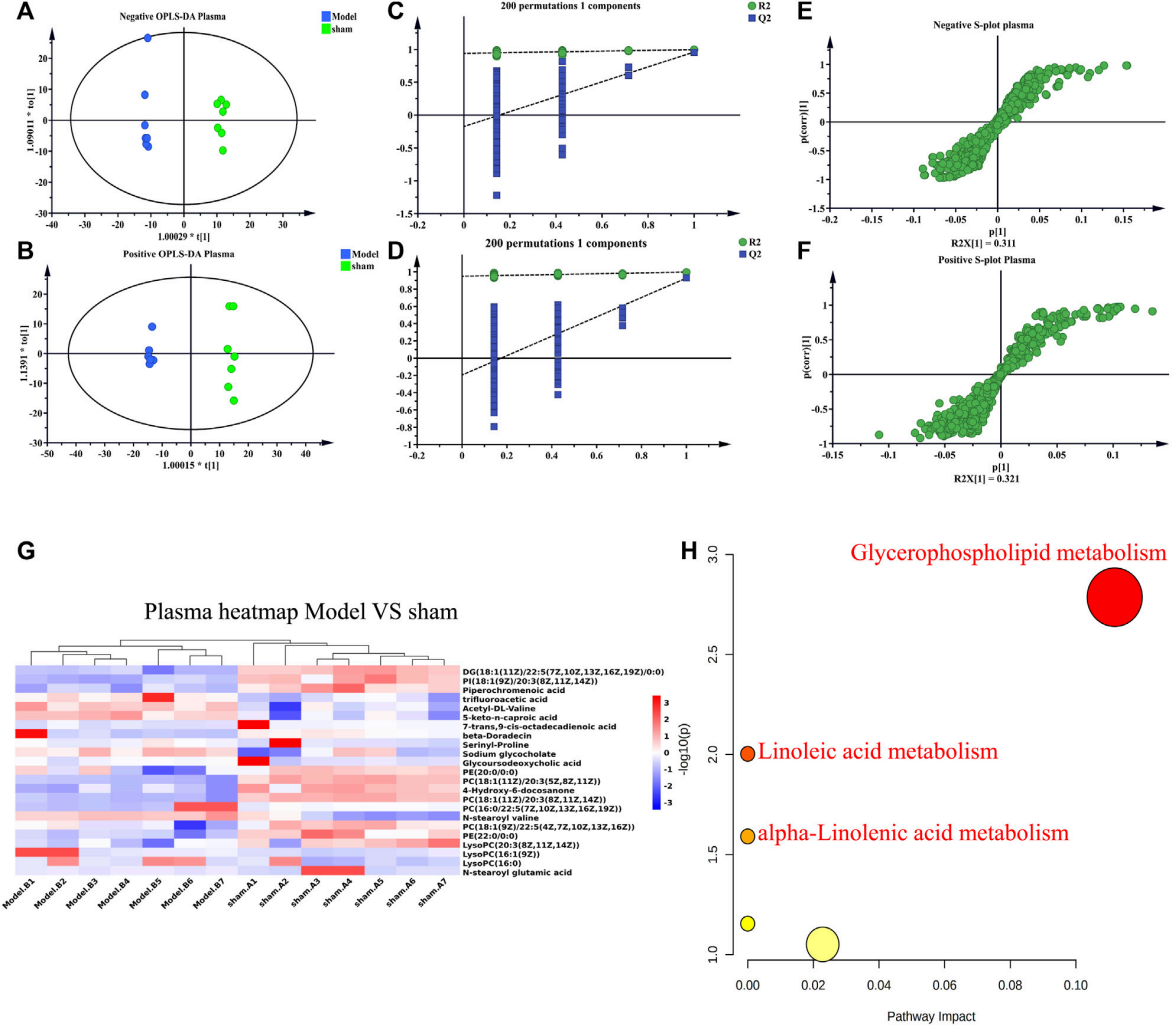
Multivariate statistical analysis of plasma in sham and model groups of rats. **(A–B)** OPLS-DA score plots of sham and model groups. **(C–D)** Validation plots after 200 replacement tests. **(E–F)** S-plots of sham and model groups from the OPLS-DA model. **(G)** Heatmap of chronic heart failure rats. **(H)** Plots of metabolic pathways associated with chronic heart failure rats. **(A, C, E)** negative mode; **(B, D, F)** positive mode.

**FIGURE 6:**
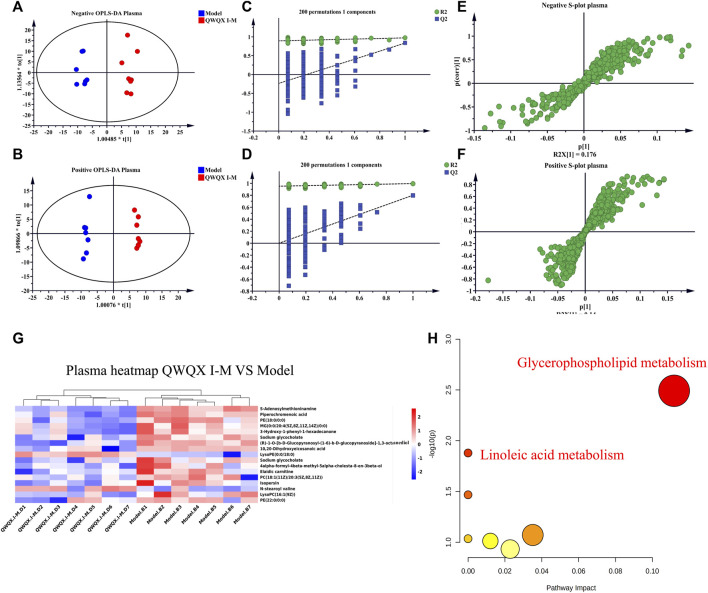
Multivariate statistical analysis of plasma in QWQX І and the model groups of rats. **(A–B)** Plots of OPLS-DA scores in QWQX І and model groups. **(C–D)** Validation plots after 200 replacement tests. **(E–F)** S-plots of QWQX І and model groups in OPLS-DA model. **(G)** Heatmap of QWQX І against chronic heart failure in rats. **(H)** Plots of metabolic pathways associated with QWQX І against chronic heart failure in rats. **(A, C, E)** negative mode; **(B, D, F)** positive mode.

UHPLC-Q-TOF-MS was used for data acquisition of plasma samples, and the total ion flow chromatograms of rat plasma in positive and negative ion modes (Supplementary Figure S1). Plasma quality control samples showed tight clustering results in the PCA score plot in positive and negative ion mode, and PCA also showed that all quality control samples are within 2 times the standard deviation (SD) in the score plot, which means the stability of the UHPLC-QTOF/MS system in batch analysis was satisfactory (Supplementary Figure S1). Multivariate statistical analysis constructed an unsupervised and integrated view of OPLS-DA to explore distributions and trends in the sham, Model, and QWQX І groups. As shown in Figures 4A, B, the three groups had a good trend of separation in 3D space, indicating that significant metabolic changes occurred after LAD induction. An OPLS-DA model was performed to show pairwise differences between the sham, Model, and QWQX І groups to explore potential differential metabolites associated with chronic heart failure and QWQX І treatment. Figures 5A, B showed good separation between the sham and Model groups in the OPLS-DA score plots, revealing significant changes in metabolites in LAD-induced chronic heart failure rats; Figures 6A, B showed that Model group and QWQX І group rat plasma samples were clearly separated in the supervised OPLS-DA score map in positive and negative ion mode, indicating that QWQX І has a certain regulatory effect on the rat plasma metabolic disorder caused by chronic heart failure. Furthermore, 200 times permutation tests were performed to validate the OPLS-DA model and to avoid overfitting, resulting in a pattern with intercepts of R2 = 0.994, Q2 = -0.0913 and R2 = 0.994, Q2 = -0.0913, indicating that the established OPLS-DA model has outstanding applicability and predictability (Figures 5C, D and Figures 6C, D). Metabolites were carefully screened before being approved as potential differential metabolites. A visual sigmoid plot was used to show the relationship between the covariance and correlation of the OPLS-DA model and the variable importance (VIP) value in the projection to identify features that contributed to group separation, reducing metabolite selection. risk of false positives. As shown in Figures 5E, F and the S panel of Figures 6E, F, combined with VIP value (VIP > 1) and t-test (*p* < 0.05) and combined with online databases such as HMDB and KEGG, 23 and 16 metabolites with important contributions after CHF interference and QWQX І treatment were screened in positive and negative mode (Tables 3 and 4). A heatmap cluster analysis of their shared differential metabolites (Figures 5G, 6G) showed that the different metabolites leveled back to the sham group after QWQX І treatment. The 23 and 16 differential metabolites were imported into the MetaboAnalyst 5.0 (https://www.metaboanalyst.ca/) webpage for metabolic pathway analysis, with *p* < 0.05 or pathway impact > 0.1 as the screening criteria for potential key metabolic pathways (Figures 5H, 6H), the results show that QWQX І mainly regulates the metabolism of plasma through glycerophospholipid metabolism and linoleic acid metabolism to exert its therapeutic effect on CHF.

**TABLE 3:** Differential metabolites in rat plasma associated with chronic heart failure (*p* < 0.05).

Name	Formula	mode	Mass m/z	RT (min)	HMDB	Log_2_FC
DG (18:1 (11Z)/22:5 (7Z,10Z,13Z,16Z,19Z)/0:0)	C43 H72 O5	ESI-	668.5356	14.476	HMDB07207	-1.34
PI(18:1 (9Z)/20:3 (8Z,11Z,14Z))	C47 H83 O13 P	ESI-	886.5514	19.18	HMDB09843	-1.60
Piperochromenoic acid	C22 H28 O3	ESI-	340.2053	13.198	HMDB0040635	-0.75
trifluoroacetic acid	C2 H F3 O2	ESI-	113.9929	0.87	HMDB14118	0.59
Acetyl-DL-Valine	C7 H13 N O3	ESI-	159.0895	4.921	HMDB11757	1.97
5-keto-n-caproic acid	C6 H10 O3	ESI-	130.0628	4.526	HMDB0061881	0.80
7-trans,9-cis-octadecadienoic acid	C18 H32 O2	ESI-	280.2392	14.357	HMDB0062784	-0.79
beta-Doradecin	C40 H52 O3	ESI-	580.3938	16.188	HMDB39143	-2.47
Serinyl-Proline	C8 H14 N2 O4	ESI-	202.0947	0.999	HMDB29047	1.03
Sodium glycocholate	C26 H43 N O6	ESI-	465.3075	8.316	HMDB32596	1.31
Glycoursodeoxycholic acid	C26 H43 N O5	ESI-	449.3127	9.522	HMDB00708	1.542
PE (20:0/0:0)	C25 H52 N O7 P	ESI-	509.3465	12.88	HMDB0011511	-0.60
PC(18:1 (11Z)/20:3 (5Z,8Z,11Z))	C46 H85 N O8 P	ESI+	810.604	15.213	HMDB0008079	-3.93
4-Hydroxy-6-docosanone	C22 H44 O2	ESI+	340.3346	12.693	HMDB35667	-2.35
PC(18:1 (11Z)/20:3 (8Z,11Z,14Z))	C46 H85 N O8 P	ESI+	810.6043	15.241	HMDB08080	-4.72
PC(16:0/22:5 (7Z,10Z,13Z,16Z,19Z))	C46 H83 N O8 P	ESI+	808.5871	18.15	HMDB07990	-3.25
N-stearoyl valine	C23 H45 N O3	ESI+	383.3399	15.008	HMDB0241952	1.49
PC(18:1 (9Z)/22:5 (4Z,7Z,10Z,13Z,16Z))	C48 H85 N O8 P	ESI+	834.6027	15.183	HMDB08121	-1.26
PE (22:0/0:0)	C27 H56 N O7 P	ESI+	537.3799	14.087	HMDB0011520	-1.01
LysoPC(20:3 (8Z,11Z,14Z))	C28 H53 N O7 P	ESI+	546.3579	12.258	HMDB10394	-0.59
LysoPC(16:1 (9Z))	C24 H49 N O7 P	ESI+	494.3247	11.2	HMDB0010383	0.95
LysoPC(16:0)	C24 H51 N O7 P	ESI+	496.3422	12.198	HMDB0010382	3.04
N-stearoyl glutamic acid	C23 H43 N O5	ESI+	413.3136	13.18	HMDB0241942	1.20

**TABLE 4:** Differential metabolites of QWQX І anti-chronic heart failure-related rat plasma (*p* < 0.05).

Name	Formula	mode	Mass m/z	RT (min)	HMDB	Log_2_FC
S-Adenosylmethioninamine	C14 H23 N6 O3 S	ESI-	355.158	14.988	HMDB00988	-1.11
Piperochromenoic acid	C22 H28 O3	ESI-	340.2049	13.295	HMDB40635	-0.58
PE (18:0/0:0)	C23 H48 N O7 P	ESI-	481.3148	12.214	HMDB0011130	-0.35
MG (0:0/20:4 (5Z,8Z,11Z,14Z)/0:0)	C23 H38 O4	ESI-	378.2777	14.36	HMDB0004666	-0.32
3-Hydroxy-1-phenyl-1-hexadecanone	C22 H36 O2	ESI-	332.2727	14.976	HMDB35677	-0.34
Sodium glycocholate	C26 H43 N O6	ESI-	465.3075	8.316	HMDB32596	-1.10
(R)-1-O-[b-D-Glucopyranosyl-(1–6)-b-D-glucopyranoside]-1,3-octanediol	C20 H38 O12	ESI+	470.237	6.271	HMDB0032799	-0.41
10,20-Dihydroxyeicosanoic acid	C20 H40 O4	ESI+	344.2932	9.53	HMDB31923	-1.12
LysoPE (0:0/18:0)	C23 H48 N O7 P	ESI+	481.3139	5.708	HMDB11129	0.40
Sodium glycocholate	C26 H43 N O6	ESI+	465.3099	8.399	HMDB32596	-1.08
4alpha-formyl-4beta-methyl-5alpha-cholesta-8-en-3beta-ol	C29 H48 O2	ESI+	428.3663	18.357	HMDB0012168	-0.39
Elaidic carnitine	C25 H48 N O4	ESI+	426.3586	12.379	HMDB06464	-0.59]
PC(18:1 (11Z)/20:3 (5Z,8Z,11Z))	C46 H85 N O8 P	ESI+	810.6044	15.24	HMDB0008079	-0.40]
Isopersin	C23 H40 O4	ESI+	380.2919	11.425	HMDB32735	-0.40
N-stearoyl valine	C23 H45 N O3	ESI+	383.3399	15.071	HMDB0241952	0.90
LysoPC(16:1 (9Z))	C24 H49 N O7 P	ESI+	494.3267	11.365	HMDB10383	-0.31
PE (22:0/0:0)	C27 H56 N O7 P	ESI+	537.3799	14.087	HMDB0011520	-0.47

### 3.5 QWQX І modulated the metabolome of cardiac tissue in CHF rats

**FIGURE 7:**
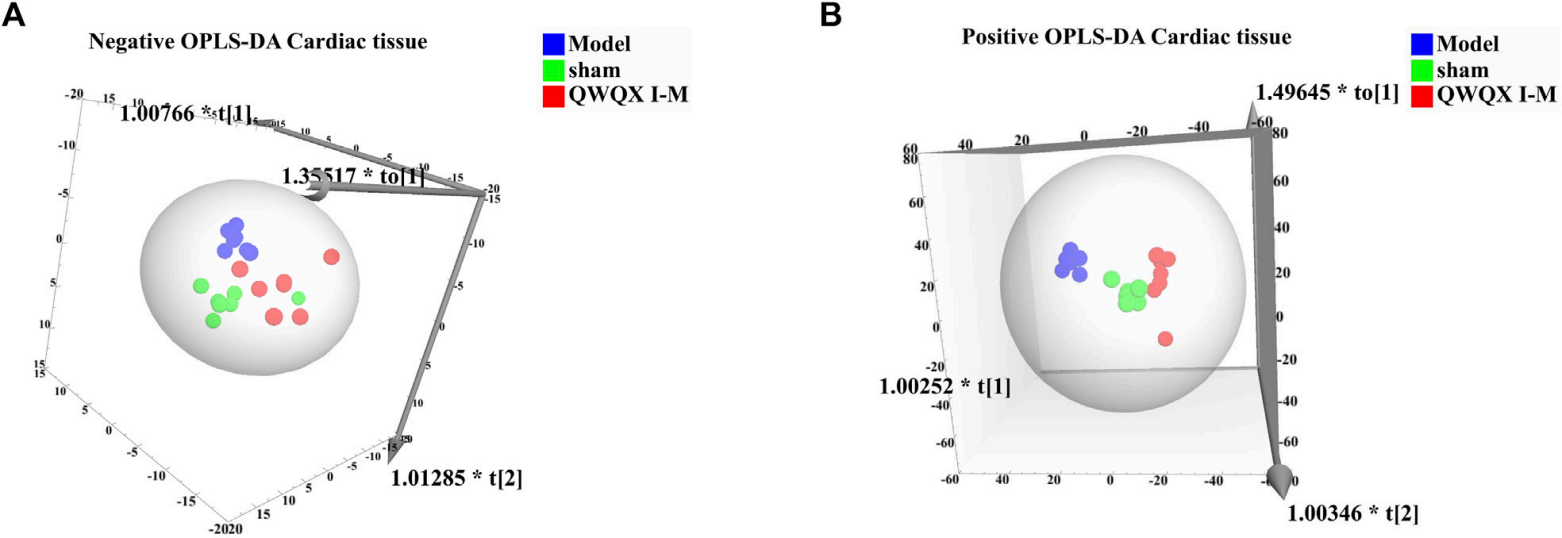
Cardiac tissue metabolomics OPLS-DA scores for each group of rats. **(A)** Negative mode of various rats. **(B)** Positive mode of various rats.

**FIGURE 8:**
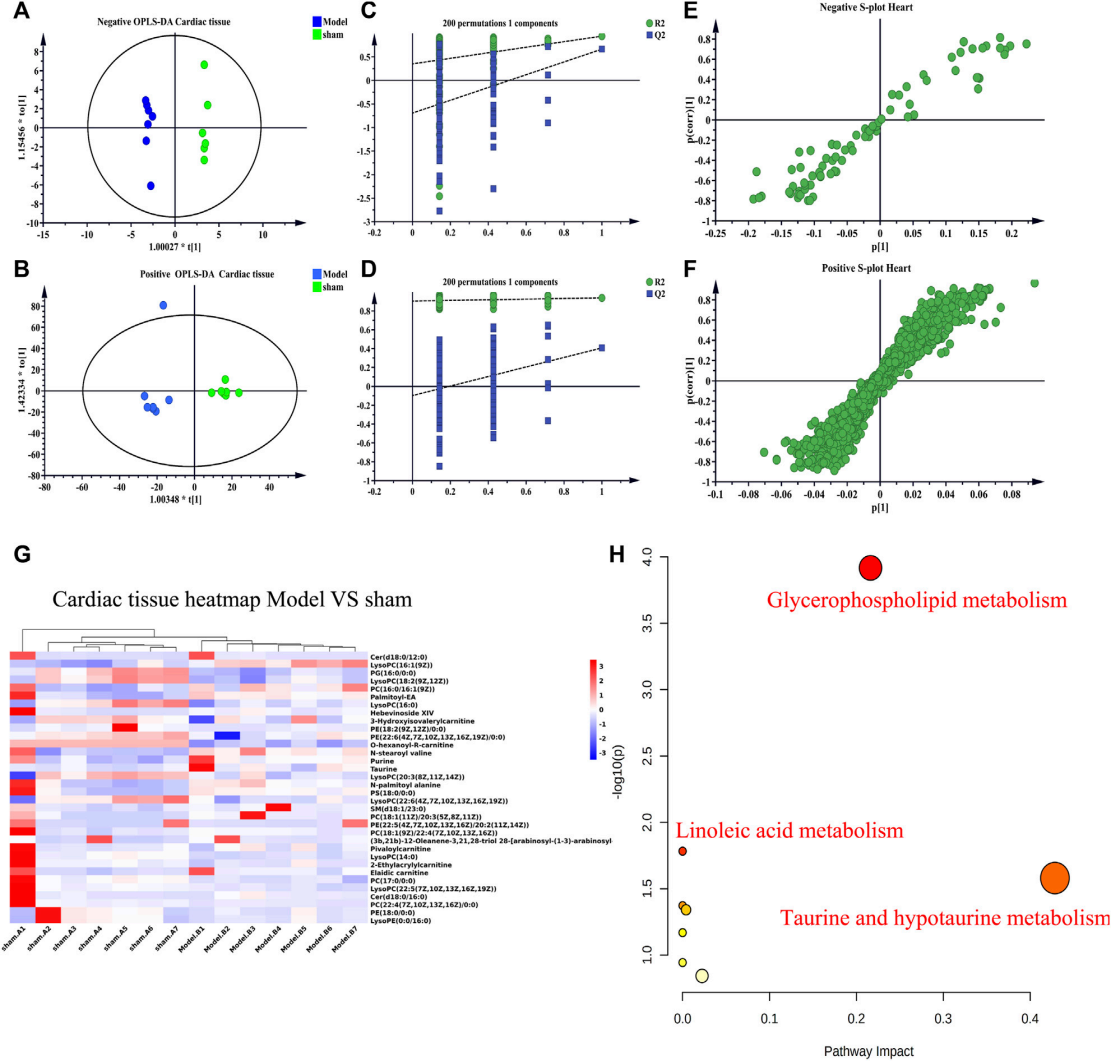
Multivariate statistical analysis of cardiac tissue in sham and model groups of rats. **(A–B)** OPLS-DA score plots of sham and model groups. **(C–D)** validation plots after 200 replacement tests. **(E–F)** S-plots of sham and model groups from the OPLS-DA model. **(G)** Heatmap of chronic heart failure rats. **(H)** plots of metabolic pathways associated with chronic heart failure rats. **(A, C, E)** negative mode; **(B, D, F)** positive mode.

**FIGURE 9:**
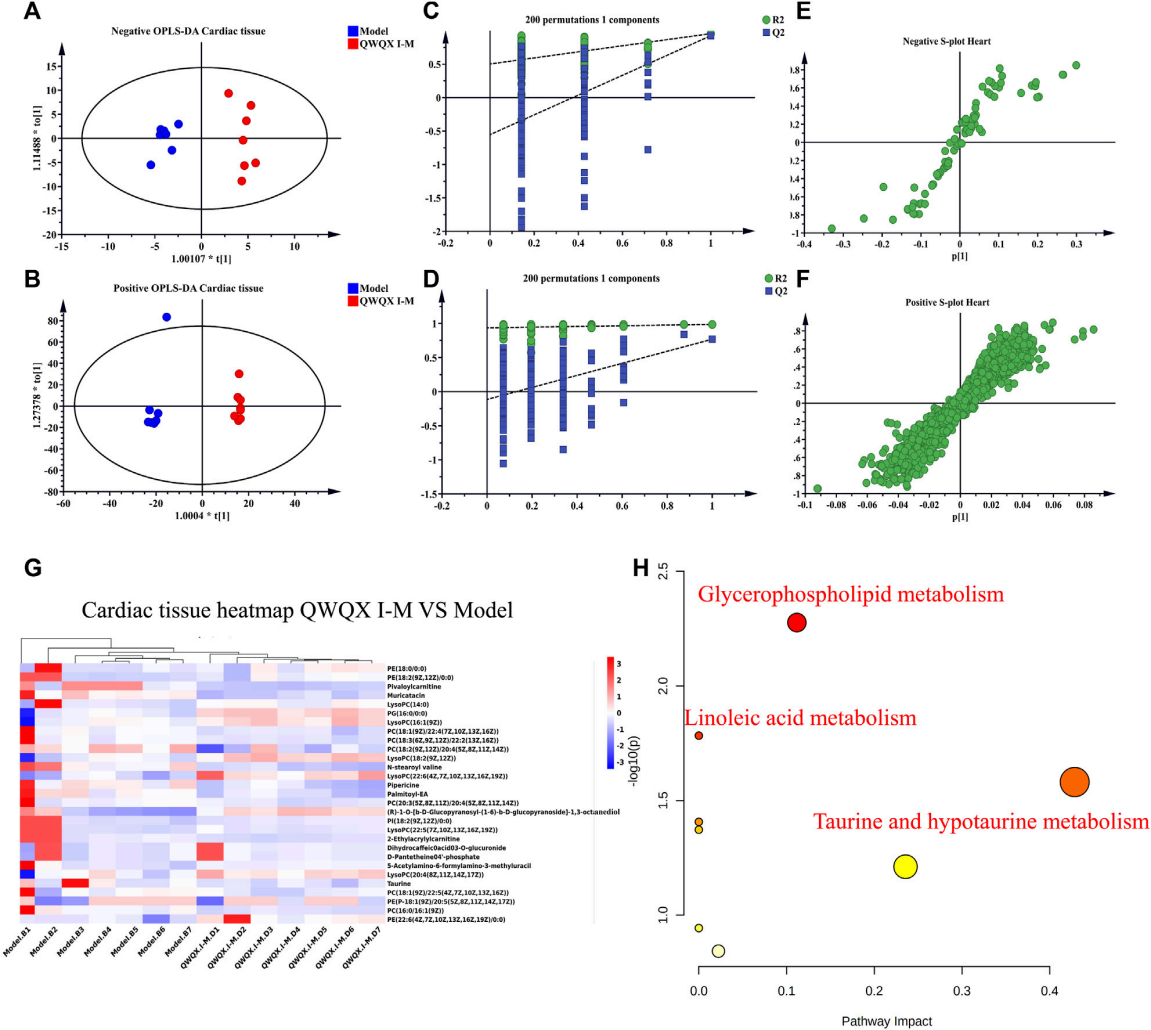
Multivariate statistical analysis of cardiac tissue in QWQX І and model groups of rats. **(A–B)** Plots of OPLS-DA scores in QWQX І and model groups. **(C–D)** Validation plots after 200 replacement tests. **(E–F)** S-plots of QWQX І and model groups in OPLS-DA model. **(G)** Heatmap of QWQX І against chronic heart failure in rats, **(H)** Plots of metabolic pathways associated with QWQX І against chronic heart failure in rats. **(A, C, E)** negative mode; **(B, D, F)** positive mode.

UHPLC-Q-TOF-MS was to collect data on cardiac tissue samples, and the total ion flow chromatograms of rat cardiac tissue in positive and negative ion modes are shown in Supplementary Figure S2. The PCA score plots of cardiac tissue QC samples in positive and negative ion mode showed tight clustering results, and the PCA also showed that all QC samples are within 2 times the SD of the score plot, which means the stability of the UHPLC-Q-TOF-MS system in batch analysis was satisfactory (Supplementary Figure S2). Multivariate statistical analysis constructed an unsupervised and integrated view of OPLS-DA to explore distributions and trends in the sham, Model, and QWQX І groups. As shown in Figures 7A, B, the three groups had a good trend of separation in 3D space, indicating that significant metabolic changes occurred after LAD induction. An OPLS-DA model was performed to show pairwise differences between the sham, Model, and QWQX І groups to explore potential differential metabolites associated with chronic heart failure and QWQX І treatment. Figures 8A, B show good separation between the sham and Model groups in the OPLS-DA score plot, revealing significant changes in metabolites in LAD-induced chronic heart failure rats; Figures 9A, B show, Model group and QWQX І group rat plasma samples were clearly separated in the supervised OPLS-DA score map in positive and negative ion mode, indicating that QWQX І has a certain regulatory effect on the metabolic disorder of rat cardiac tissue caused by chronic heart failure. In addition, to validate the OPLS-DA model and to avoid overfitting, performed 200 times permutation tests, and high values of Q2 were obtained without overfitting, indicating the outstanding applicability and predictiveness of the established OPLS-DA model (Figures 8C, D and Figures 9C, D). Metabolites were carefully screened before being approved as potential differential metabolites. A visual sigmoid plot was used to show the relationship between the covariance and correlation of the OPLS-DA model and the variable importance (VIP) value in the projection to identify features that contributed to group separation, reducing metabolite selection. risk of false positives. As shown in Figure 8 E-F and 9 E-F’s diagram, combined with VIP value (VIP>1) and t-test (*p* < 0.05) and combined with online databases such as HMDB and KEGG, the metabolites that contributed significantly after obtaining CHF and QWQX І treatment in positive and negative mode were screened34 and 32 (Tables 5 and 6). Heatmap clustering analysis of their shared differential metabolites (Figure 8G; Figure 9G) showed that different metabolites were leveled back to the sham group after QWQX І treatment. The 34 and 32 differential metabolites were imported into the MetaboAnalyst 5.0 (https://www.metaboanalyst.ca/) webpage for metabolic pathway analysis, with *p* < 0.05 or pathway impact > 0.1 as the screening criteria for potential key metabolic pathways (Figure 8H; Figure 9H), the results show that QWQX І mainly regulates the metabolism of cardiac tissue through taurine and hypotaurine metabolism, glycerophospholipid metabolism and linoleic acid metabolism to exert its therapeutic effect on CHF.

**TABLE 5:** Differential metabolites in rat cardiac tissue associated with chronic heart failure (*p* < 0.05).

Name	Formula	mode	Mass m/z	RT (min)	HMDB	Log_2_FC
PE (18:0/0:0)	C23 H48 N O7 P	ESI-	481.3162	13.608	HMDB0011130	-1.18
LysoPE (0:0/16:0)	C21 H44 N O7 P	ESI-	453.2851	12.294	HMDB0011473	-1.16
Cer(d18:0/12:0)	C30 H61 N O3	ESI+	483.4619	14.274	HMDB11758	4.81
LysoPC(16:1 (9Z))	C24 H49 N O7 P	ESI+	494.3245	11.434	HMDB0010383	-2.75
PG (16:0/0:0)	C22 H45 O9 P	ESI+	484.2803	13.286	HMDB0240601	-2.32
LysoPC(18:2 (9Z,12Z))	C26 H51 N O7 P	ESI+	520.3423	11.778	HMDB10386	-1.87
PC(16:0/16:1 (9Z))	C40 H79 N O8 P	ESI+	732.5535	18.461	HMDB0007969	1.18
Palmitoyl-EA	C18 H37 N O2	ESI+	299.2826	11.993	HMDB02100	1.32
LysoPC(16:0)	C24 H51 N O7 P	ESI+	496.3417	12.058	HMDB0010382	-1.20
Hebevinoside XIV	C47 H74 O15	ESI+	878.5048	17.466	HMDB0034604	3.21
3-Hydroxyisovalerylcarnitine	C12 H24 N O5	ESI+	262.1652	2.644	HMDB0061189	-1.67
PE (18:2 (9Z,12Z)/0:0)	C23 H44 N O7 P	ESI+	477.2871	11.499	HMDB0011507	-2.20
PE (22:6 (4Z,7Z,10Z,13Z,16Z,19Z)/0:0)	C27 H44 N O7 P	ESI+	525.2873	11.466	HMDB0011526	-0.77
O-hexanoyl-R-carnitine	C13 H26 N O4	ESI+	260.1861	5.557	HMDB00756	-13.02
N-stearoyl valine	C23 H45 N O3	ESI+	383.3409	15.012	HMDB0241952	1.29
Purine	C5 H4 N4	ESI+	120.0437	9.315	HMDB01366	0.91
Taurine	C2 H7 N O3 S	ESI+	125.015	0.912	HMDB0000251	4.06
LysoPC(20:3 (8Z,11Z,14Z))	C28 H53 N O7 P	ESI+	546.3558	12.016	HMDB0010394	-1.77
N-palmitoyl alanine	C19 H37 N O3	ESI+	327.2774	13.227	HMDB0241919	1.52
PS(18:0/0:0)	C24 H48 N O9 P	ESI+	525.3049	13.287	HMDB0240606	0.96
LysoPC(22:6 (4Z,7Z,10Z,13Z,16Z,19Z))	C30 H51 N O7 P	ESI+	568.3419	11.68	HMDB0010404	-1.14
SM(d18:1/23:0)	C46 H94 N2 O6 P	ESI+	801.6831	19.159	HMDB0012105	3.36
PC(18:1 (11Z)/20:3 (5Z,8Z,11Z))	C46 H85 N O8 P	ESI+	810.6028	13.795	HMDB0008079	2.48
PE (22:5 (4Z,7Z,10Z,13Z,16Z)/20:2 (11Z,14Z))	C47 H80 N O8 P	ESI+	817.5621	18.517	HMDB09627	2.33
PC(18:1 (9Z)/22:4 (7Z,10Z,13Z,16Z))	C48 H87 N O8 P	ESI+	836.6163	18.651	HMDB0008120	2.00
(3b,21b)-12-Oleanene-3,21,28-triol 28-[arabinosyl-(1–3)-arabinosyl-(1–3)-arabinoside]	C45 H74 O15	ESI+	854.5047	17.862	HMDB0033641	1.57
Pivaloylcarnitine	C12 H24 N O4	ESI+	246.1708	4.376	HMDB41993	7.16
LysoPC(14:0)	C22 H47 N O7 P	ESI+	468.309	10.991	HMDB10379	0.50
2-Ethylacrylylcarnitine	C12 H22 N O4	ESI+	244.1549	4.116	HMDB0240764	0.73
Elaidic carnitine	C25 H48 N O4	ESI+	426.3591	12.318	HMDB0006464	4.77
PC(17:0/0:0)	C25 H53 N O7 P	ESI+	510.3574	12.99	HMDB0012108	0.26
LysoPC(22:5 (7Z,10Z,13Z,16Z,19Z))	C30 H53 N O7 P	ESI+	570.3558	11.817	HMDB0010403	0.86
Cer(d18:0/16:0)	C34 H69 N O3	ESI+	539.5283	15.236	HMDB0011760	2.49
PC(22:4 (7Z,10Z,13Z,16Z)/0:0)	C30 H55 N O7 P	ESI+	572.3714	12.503	HMDB0010401	1.40

**TABLE 6:** Differential metabolites of QWQX І anti-chronic heart failure-related rat cardiac tissue (*p* < 0.05).

Name	Formula	mode	Mass m/z	RT (min)	HMDB	Log_2_FC
PE (18:0/0:0)	C23 H48 N O7 P	ESI-	481.3162	13.608	HMDB0011130	1.22
PE (P-18:1 (9Z)/20:5 (5Z,8Z,11Z,14Z,17Z))	C43 H74 N O7 P	ESI-	747.5179	19.152	HMDB11453	4.11
PE (18:2 (9Z,12Z)/0:0)	C23 H44 N O7 P	ESI-	477.2852	11.738	HMDB0011507	1.55
Pivaloylcarnitine	C12 H24 N O4	ESI+	246.1708	4.376	HMDB41993	-9.07
Muricatacin	C17 H32 O3	ESI+	284.2351	9.707	HMDB38685	-1.01
LysoPC(14:0)	C22 H47 N O7 P	ESI+	468.3093	12.957	HMDB0010379	3.45
PE (22:6 (4Z,7Z,10Z,13Z,16Z,19Z)/0:0)	C27 H44 N O7 P	ESI+	525.2873	11.644	HMDB0011526	1.34
PE (P-18:1 (9Z)/20:5 (5Z,8Z,11Z,14Z,17Z))	C43 H74 N O7 P	ESI+	747.5202	18.044	HMDB11453	-1.06
PG (16:0/0:0)	C22 H45 O9 P	ESI+	484.2803	13.286	HMDB0240601	1.83
LysoPC(16:1 (9Z))	C24 H49 N O7 P	ESI+	494.3245	11.434	HMDB0010383	1.84
PC(18:1 (9Z)/22:4 (7Z,10Z,13Z,16Z))	C48 H87 N O8 P	ESI+	836.6163	18.651	HMDB0008120	-2.18
PC(18:3 (6Z,9Z,12Z)/22:2 (13Z,16Z))	C48 H87 N O8 P	ESI+	836.6153	18.65	HMDB0008185	-2.02
PC(18:2 (9Z,12Z)/20:4 (5Z,8Z,11Z,14Z))	C46 H81 N O8 P	ESI+	806.5688	18.48	HMDB0008147	-1.22
LysoPC(18:2 (9Z,12Z))	C26 H51 N O7 P	ESI+	520.3423	11.778	HMDB10386	1.51
N-stearoyl valine	C23 H45 N O3	ESI+	383.3409	15.012	HMDB0241952	-1.04
LysoPC(22:6 (4Z,7Z,10Z,13Z,16Z,19Z))	C30 H51 N O7 P	ESI+	568.3419	11.68	HMDB0010404	1.44
PC(16:0/16:1 (9Z))	C40 H79 N O8 P	ESI+	732.5545	18.128	HMDB0007969	-2.61
Pipericine	C22 H41 N O	ESI+	335.3186	13.495	HMDB0031678	-0.94
Palmitoyl-EA	C18 H37 N O2	ESI+	299.2826	11.993	HMDB02100	-0.84
PC(20:3 (5Z,8Z,11Z)/20:4 (5Z,8Z,11Z,14Z))	C48 H83 N O8 P	ESI+	832.5864	17.352	HMDB0008378	-2.09
(R)-1-O-[b-D-Glucopyranosyl-(1–6)-b-D-glucopyranoside]-1,3-octanediol	C20 H38 O12	ESI+	470.2358	6.258	HMDB0032799	0.97
PI(18:2 (9Z,12Z)/0:0)	C27 H49 O12 P	ESI+	596.2962	11.38	HMDB0240597	0.53
LysoPC(22:5 (7Z,10Z,13Z,16Z,19Z))	C30 H53 N O7 P	ESI+	570.356	12.014	HMDB0010403	0.57
2-Ethylacrylylcarnitine	C12 H22 N O4	ESI+	244.1549	4.116	HMDB0240764	-1.11
Dihydrocaffeic acid 3-O-glucuronide	C15 H18 O10	ESI+	358.0912	3.265	HMDB41720	4.49
D-Pantetheine 4′-phosphate	C11 H23 N2 O7 P S	ESI+	358.0959	3.265	HMDB0001416	4.43
5-Acetylamino-6-formylamino-3-methyluracil	C8 H10 N4 O4	ESI+	226.0715	3.703	HMDB11105	-1.15
LysoPC(20:4 (8Z,11Z,14Z,17Z))	C28 H51 N O7 P	ESI+	544.342	11.756	HMDB0010396	1.20
PC(16:0/16:1 (9Z))	C40 H79 N O8 P	ESI+	732.5535	18.461	HMDB0007969	-0.95
PE (22:6 (4Z,7Z,10Z,13Z,16Z,19Z)/0:0)	C27 H44 N O7 P	ESI+	525.2873	11.644	HMDB0011526	3.02
Taurine	C2 H7 N O3 S	ESI+	125.015	0.912	HMDB0000251	-3.35
PC(18:1 (9Z)/22:5 (4Z,7Z,10Z,13Z,16Z))	C48 H85 N O8 P	ESI+	834.6037	18.41	HMDB0008121	-1.88

### 3.6 Metabolic pathway analysis

**FIGURE 10:**
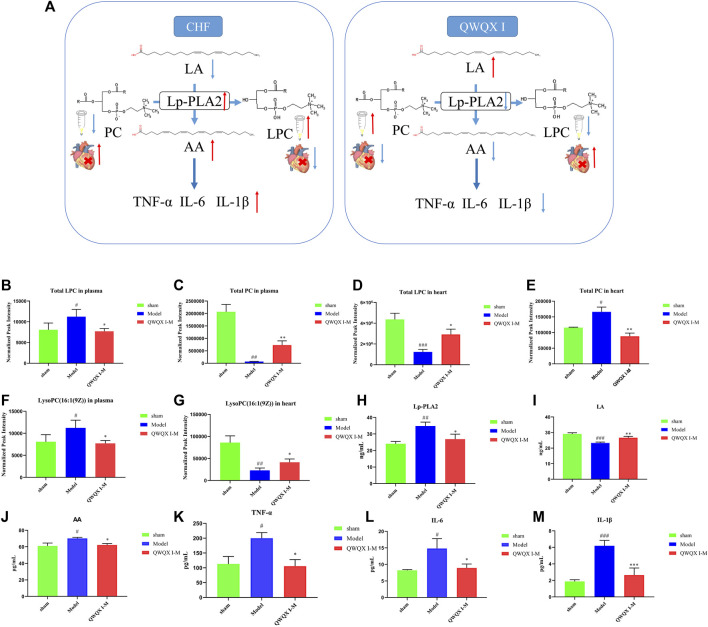
Screening of plasma and cardiac tissue candidate key differential metabolites in CHF rats after treatment with QWQX І. **(A)** Potential metabolic pathways. **(B)** Total LPC in plasma. **(C)** Total PC in plasma. **(D)** Total LPC in heart tissue. **(E)** Total PC in heart tissue. **(F)** LysoPC(16:1 (9Z)) in plasma. **(G)** LysoPC(16:1 (9Z)) in heart tissue. **(H)** The level of Lp-PLA2. **(I)** The level of AA. **(J)** The level of LA. **(K)** The level of TNF- α. **(L)** The level of IL-6. **(M)** The level of IL-1β. Data are expressed as the Mean ± SEM. ^#^ *p* < 0.05, ^##^ *p* < 0.01, ^###^ *p* < 0.001, the Model group vs. the sham group; *p < 0.05, **p < 0.01, ***p < 0.001, drug treatment group vs. the Model group.

The top three enriched metabolic pathways affected by QWQX І included taurine and hypotaurine metabolism, glycerophospholipid metabolism and linoleic acid metabolism. Notably, these metabolic pathways are interconnected and QWQX І may affect these different therapeutic pathways for the purpose of treating CHF. To investigate the working mechanism of QWQX І, the KEGG (Kyoto Encyclopedia of Genes and Genomes) database and related metabolites were used as summarized in Figure 10A. Lp-PLA2 has the function of catalyzing the hydrolysis of phosphatidylcholine (PC) to lysophosphatidylcholine (LPC), so we compared the semiquantitative plasma and cardiac peak intensity levels of PC and LPC between different groups (Figures 10B–E). We found that the expression levels of PC and LPC in plasma and heart were opposite in CHF state and after QWQX І treatment, which should be caused by the release of LPC-like substances into the blood after heart failure. Notably, a key differential metabolite LysoPC(16:1 (9Z)) shared by plasma and heart was produced by Lp-PLA2 (Figures 10F, G), and we detected the enzyme activity for Lp-PLA2 by ELISA kit. As shown in Figure 10H, the activity of Lp-PLA2 was increased in the model group (*p* < 0.01), while QWQX І treatment could reduce that (*p* < 0.05). Since Lp-PLA2 can hydrolyze oxidized linoleic acid to produce pro-inflammatory substances, we also used ELISA kits to detect linoleic acid (LA), arachidonic acid (AA), tumor necrosis factor-α (TNF-α), leukocytes Interleukin 6 (IL-6) and Interleukin 1β (IL-1β). As shown in Figure 10I–M, the expression levels of pro-inflammatory substances in the model group increased to varying degrees (*p* < 0.05), and returned to normal levels after treatment with QWQX І. These results suggest that QWQX І reverses the LAD-induced cardiac remodeling process in CHF rats by suppressing the levels of inflammatory factors TNF-α, IL-1β, and IL-6 in LAD-induced CHF through mediating Lp-PLA2.

## 4 Discussion

In this study, QWQX І was proved to be effective against MI-induced CHF. Clinical studies, *in vivo* pharmacodynamics and metabolomics revealed the following findings: 1) QWQX І improved cardiac function and cardiac remodeling; 2) metabolite enrichment analysis suggested that glycerophospholipid metabolism and taurine and hypotaurine metabolism were the potential pathways for CHF therapy of QWQX I; 3) KEGG analysis showed that Lp-PLA2 is a key metabolic enzyme regulated by QWQX I in CHF.

TCM have advocated combination therapy to treat cardiovascular diseases, such as heart failure (Zhang et al., 2019). QWQX І is an experienced prescription based on the theory of traditional Chinese medicine, which includes 7 herbs. Among them, astragalus and ginseng are the main herbs with positive inotropic, positive chronotropic, vasodilatory, anti-inflammatory and diuretic effects in myocardial ischemia and failure (He et al., 2021). Ginseng inhibits cardiomyocyte hypertrophy and heart failure by inhibiting Na^+^-H^+^ exchanger 1 (NHE-1) and attenuating calcineurin activation (Guo et al., 2011). At the same time, ginseng can reverse the established cardiomyocyte hypertrophy and heart failure after myocardial infarction (Moey et al., 2012). Huangqi granule protects failing hearts against electrical remodeling by downregulating CaMKII (Li et al., 2017). In the present study, the clinical trial suggested that QWQX І improved the cardiac function and quality of life in patients with heart failure. *In vivo* animal study also showed that QWQX І could improve myocardial function, reduce left ventricular size and inhibit interstitial inflammation and fibrosis.

Studying the mechanism of action of drugs is one of the most important tasks in drug research. Since QWQX І has shown positive clinical effects in the treatment of heart failure, we used metabolomics analysis to explore possible molecular mechanism of QWQX І against CHF. It was found that QWQX І could regulate the metabolism of taurine and hypotaurine, glycerophospholipid metabolism and linoleic acid metabolism pathways. Taurine is a key metabolite in the metabolic pathway of taurine and hypotaurine, and ubiquitously present in the mitochondria of cardiomyocytes (Schaffer et al., 2016) to maintain the normal respiratory chain of mitochondria. Taurine intake can increase the activity of mitochondrial carnitine palmitoyltransferase (CPT) involved in fatty acid oxidation and ketone body production in rats (Murakami et al., 2018). Taurine is mainly produced by a series of enzymatic reactions from methionine and cysteine. Cysteine promotes taurine production (Huang et al., 2019), Cysteine sulfite carboxylase that synthesizes taurine in humans (CSAD) is considered to be the rate-limiting enzyme in mammalian taurine biosynthesis (Abbasian et al., 2021), Cysteine can synthesize glutathione with glycine and glutamic acid in the body (Forman et al., 2009). Our results showed that the level of taurine in the heart of the CHF model rats was increased, while decreased after QWQX І intervention.

LysoPC(16:1 (9Z)) is a metabolite on the metabolic pathway of glycerophospholipid metabolism and co-differential metabolite in the plasma and cardiac tissue metabolome, involved in the regulation of many cellular processes. In this study, LysoPC(16:1(9Z)) level was decreased in the heart of the Model group, and its level was back to normal levels after the administration of QWQX І for intervention. Plasma LysoPC(16:1 (9Z)) was significantly higher in the Model group rats than in the sham group rats, and it was significantly reduced after administration of QWQX І. It is speculated that this may be caused by the release of LPC-like substances into the blood after the occurrence of MI. In the MI heart, the ischemic myocardium consumes more oxygen than it supplies and energy production is impaired. As hypoxia progresses and the stress state persists, the myocardium requires more energy, at which point fat mobilization increases compensatorily (Mahat et al., 2021). It has been found that the lysophospholipid content in the peripheral blood of rats with heart failure is elevated, which may be due to the fact that in heart failure, the body is in a state of chronic hypoxia, resulting in the inability of some body cells to compensate, leading to apoptosis or necrosis, and the abnormal homeostasis of lysophospholipids alters the interaction of membrane-associated protein complexes that regulate myocardial metabolism (Fu et al., 2019). PC is involved in VLDL secretion (Rinella et al., 2008) and HDL metabolism (Jacobs et al., 2004). After being secreted into the blood stream, PC on lipoprotein particles is degraded at the Sn-2 site of oxidized fatty acids by hydrolysis of Lp-PLA 2, which then produces LPC under various physiological and pathological conditions (Law et al., 2019). LysoPC(16:1 (9Z)), which is composed of palmitoleic acid chains located at the c-1 position, is associated with oxidative damage and inflammatory responses, and can cause strong coronary artery constriction, increase myocardial oxygen consumption, and expand the extent of myocardial infarction. The breakdown of fatty acids contributes to the consumption of PC, phosphatidylethanolamine (PE) and other substances (Zhu et al., 2019), leading to a decrease in its content. This reflects further deterioration of impaired lipid uptake and utilization, as well as a marker of decreased myocardial contractility. Elevated levels of lysophospholipids have been reported to induce oxidative stress in endothelial cells, leading to atherosclerosis and cardiovascular disease (Kim et al., 2009). Lyso PC is a strong pro-inflammatory mediator that releases AA through the activation of Lp-PLA2 (Zhao et al., 2018), while AA is a precursor of various bioactive substances, which can be converted into leukotrienes under the action of lipoxygenase and dehydratase, and participating in the regulation of inflammatory response and immune system, etc, (Zimmer et al., 2004). Linoleic acid (LA) is an n-6 polyunsaturated fatty acid, which is one of the biomarkers of oxidative stress (Asselin et al., 2014), and higher levels of LA can promote fatty acid metabolism in cardiomyocytes, which plays an important role in the inhibition of cardiomyocyte hypertrophy (Sun et al., 2021). Research has shown that Shengmai Yin formula exerts cardioprotective effects on rats with chronic heart failure *via* regulating Linoleic Acid metabolism (Wang et al., 2022). Therefore, we infer that QWQX І can regulate the inflammatory response, immune system regulation and compensatory increase of fat by regulating glycerophospholipid metabolism and linoleic acid metabolism disorder.

In summary, QWQX І significantly improved these biochemical parameters and metabolomic features, significantly increased plasma Lp-PLA2 levels, and decreased inflammation-related lysophosphatidic acid and mediators.

## 5 Conclusion

In this study, standard western medicine therapy combined with QWQX І further improved cardiac function in CHF patients. QWQX І can effectively relieve the symptoms of CHF model rats *via* regulating the glycerophospholipid metabolism and linoleic acid metabolism. Our findings provide a new molecular basis for the study of the intervention mechanism of QWQX І, which are helpful for further clinical applications and promotion of QWQX І in the treatment of CHF.

## Data Availability

The original contributions presented in the study are included in the article/Supplementary Material, further inquiries can be directed to the corresponding authors.

## Glossary

QWQX І: Qiweiqiangxin І granules
QLQX: Qiliqiangxin capsule
CHF: Chronic heart failure
EF: Ejection fraction
FS: Shortening fraction
LVEF: Left ventricular ejection fraction
LVESD: Left ventricular end-systolic internal diameter
LVEDD: Left ventricular end-diastolic internal diameter
LAD: Left anterior descending coronary artery
MI: Myocardial ischemia
EVS: Left ventricular end-systolic volume
EVD: Left ventricular diastolic volume
ACEI: Inhibitors of angiotensin-converting enzyme
ARBs: Inhibitors of angiotensin II receptors
ARINs: inhibitors of angiotensin receptor enkephalinase
SGLT2: sodium-glucose cotransporter 2
MLHFQ: Minnesota Cardiac Impairment Quality of Life Scale
BNP: B type natriuretic peptide
NT-proBNP: N-terminal pro-B type natriuretic peptide
HE: Hematoxylin-eosin
UHPLC-QTOF-MS: Ultra-high-performance liquid chromatography coupled with quadrupole time-of-flight mass spectrometry
PCA: Principle component analysis
OPLS-DA: Orthogonal projection to latent structures-discriminant analysis
MS: mass spectrum
ESI: Electrospray ionization
QC: Quality control
ANOVA: A one-way analysis of variance
ACEI: Inhibitors of angiotensin-converting enzyme
ARBs: Inhibitors of angiotensin II receptors
ARINs: Inhibitors of angiotensin receptor enkephalinase
SGLT2: Sodium-glucose cotransporter 2
VIP: Variable Importance in Projection
FC: fold change
TIC: Total ion chromatogram
EIC: Extracted ion chromatogram
PC: Phosphatidylcholine
LysoPC: Lysophosphatidylcholine
Lyso PE: Lysophosphatidylethanolamines
PI: Phosphatidylinositol
Lyso PA: Lysophosphatidic acid
Lp-PLA2: Lipoprotein-associated phospholipase A2
LA: Linoleic acid
AA: Arachidonic acid
TNF-α: Tumor necrosis factor-α
IL-6: Interleukin-6
IL-1β: Interleukin-1β
