# Supplementary material for: Exploring the mechanism of anti-chronic heart failure effect of qiweiqiangxin І granules based on metabolomics
Source: Front Pharmacol. 2023 Feb 13;14:1111007. doi: 10.3389/fphar.2023.1111007 (PMC9968974; doi:10.3389/fphar.2023.1111007)
Supplement: Supplementary file 1 [file Table1.DOCX]

**Supplementary material**

**Exploring the mechanism of anti-chronic heart failure effect of Qiweiqiangxin І granules based on metabolomics**

**Wanru Zhong^1†^, Yihua Li^3†^, Haixiang Zhong^1^, Yuanyuan Cheng^1^, Qi Chen^1,2^, Xinjun Zhao^2^, Zhongqiu Liu^1*^, Rong Li ^2*^, Rong Zhang ^1*^**

^1^Guangdong Provincial Key Laboratory of Translational Cancer Research of Chinese Medicines, Joint International Research Laboratory of Translational Cancer Research of Chinese Medicines, International Institute for Translational Chinese Medicine, School of Pharmaceutical Sciences, Guangzhou University of Chinese Medicine, Guangzhou, China.

^2^Department of Internal Medicine-Cardiovascular, The First Affiliated Hospital of Guangzhou University of Chinese Medicine, Guangzhou, Guangdong, China

^3^The first clinical medical college, Guangzhou University of Chinese Medicine, Guangzhou, China.

**^†^** These authors have contributed equally to this work and share first authorship

Table S1 Information on 33 characteristic compounds designated by QWQX І granules

| No. | RT/min | name | molecular formula | source |
| --- | --- | --- | --- | --- |
| 1  2  3  4  5  6  7  8  9  10  11  12  13  14  15  16  17  18  19  20  21  22  23  24  25  26  27  28  29  30  31  32  33 | 0.58  0.659  1.032  1.108  1.538  1.818  2.073  2.776  3.583  3.984  4.433  5.328  5.5  5.767  6.497  6.642  7.854  8.66  8.983  9.736  11.581  11.654  11.723  11.796  11.958  12.196  12.757  12.869  13.041  13.397  14.107  14.249  14.56 | *β*-(3,4-Dihydroxyphenyl)-D-lactic acid  Gamma-aminobutyric acid  L-Adenosine  Salviolone  Alangiside  Threonin  Dihydroisotanshinone I  Ononin  Hydroxytanshinone IIA  *β*-sitosterol  Ginsenoside La.qt  Trifolirhizin  Ethyl methoxycinnamate  Malyngamide J  Lithospermic acid  Buntansin A  Salvianolic acid G  Melitric acid B  Psuedohypericin  Dehydrouvaol  Agnosterone  Lupenone  20-Hexadecanoylingenol  Ilexoside A  21-Isopropyl-28,29,30-trinor-17,19,21-gammaceratriene  Ilexgenin A  20S-ginsenoside Rs3  Tanshinaldehyde  Perrottetin D  Isotanshinone II  Miltirone  Cryptotanshinone  Apollinine | C_9_H_10_O_5_  C_4_H_9_NO_2_  C_10_H_13_N_5_O_4_  C_18_H_20_O_2_  C_25_H_31_NO_10_  C_4_H_9_NO_3_  C_18_H_14_O_3_  C_22_H_24_O_9_  C_19_H_18_O_4_  C_30_H_52_O  C_30_H_50_O_3_  C_22_H_22_O_10_  C_12_H_14_O_3_  C_33_H_53_NO_9_  C_27_H_22_O_12_  C_11_H_8_O_5_  C_18_H_12_O_7_  C_30_H_16_O_9_  C_18_H_26_O_10_  C_19_H_20_O_3_  C_30_H_46_O  C_36_H_58_O4  C_30_H_48_O  C_35_H_56_O_8_  C_30_H_46_  C_30_H_46_O_6_  C_19_H_22_O_5_  C_19_H_16_O_4_  C_19_H_20_O_3_  C_18_H_14_O_3_  C_19_H_22_O_2_  C_19_H_22_O_3_  C_22_H_18_O_5_ | Radix Salviae  Hedysarum Multijugum Maxim.  Panax Ginseng C. A. Mey.  Radix Salviae  Cinnamomum cassia Presl  Radix Salviae  Radix Salviae  Hedysarum Multijugum Maxim.  Radix Salviae  Lepidii Semen Descurainiae Semen  Panax Ginseng C. A. Mey.  Panax Ginseng C. A. Mey.  Cinnamomi Ramulus  Radix Salviae  Radix Salviae  Radix Salviae  Radix Salviae  Panax Ginseng C. A. Mey  Radix Salviae Miltiorrhiae  Radix Salviae  Radix Ilicis Pubescentis  Panax Ginseng C. A. Mey  Astragalus membranaceus Bge  Radix Ilicis Pubescentis  Astragalus membranaceus Bge  Radix Ilicis Pubescentis  Hedysarum Multijugum Maxim.  Radix Salviae  Radix Salviae  Radix Salviae  Radix Salviae  Radix Salviae  Radix Salviae |

Table S2 Mass spectrometry conditions

| 6540 Q-TOF MS conditions | |
| --- | --- |
| Capillary voltage | 3500V |
| Gas temperature | 320℃ |
| Gas flow rate | 8 L/min |
| Nebulizer | 35 psig |
| Sheath gas temperature | 350℃ |
| Sheath gas flow rate | 1l L/min |
| Nozzle voltage | 1500V |
| Fragmentor | 175V |
| Skimmer | 65V |
| Scan rate | 4 spectra/s |

The instrument mode is Extended Mass Range (2 GHz), the acquisition mode is full scan, and the scan mass-to-charge ratio range is 50-1500, with positive mode reference ions of 121.0509 and 922.0098, and negative mode reference ions of 112.9855 and 1033.9881.

Table S3 Gradient elution conditions

| Time | Flow rate | Mobile phase A | Mobile phase B |
| --- | --- | --- | --- |
| (min) | (mL/min) | (%) | (%) |
| 0 | 0.4 | 98 | 2 |
| 1 | 0.4 | 98 | 2 |
| 15 | 0.4 | 0 | 100 |
| 18 | 0.4 | 0 | 100 |
| 18.01 | 0.4 | 98 | 2 |
| 20 | 0.4 | 98 | 2 |


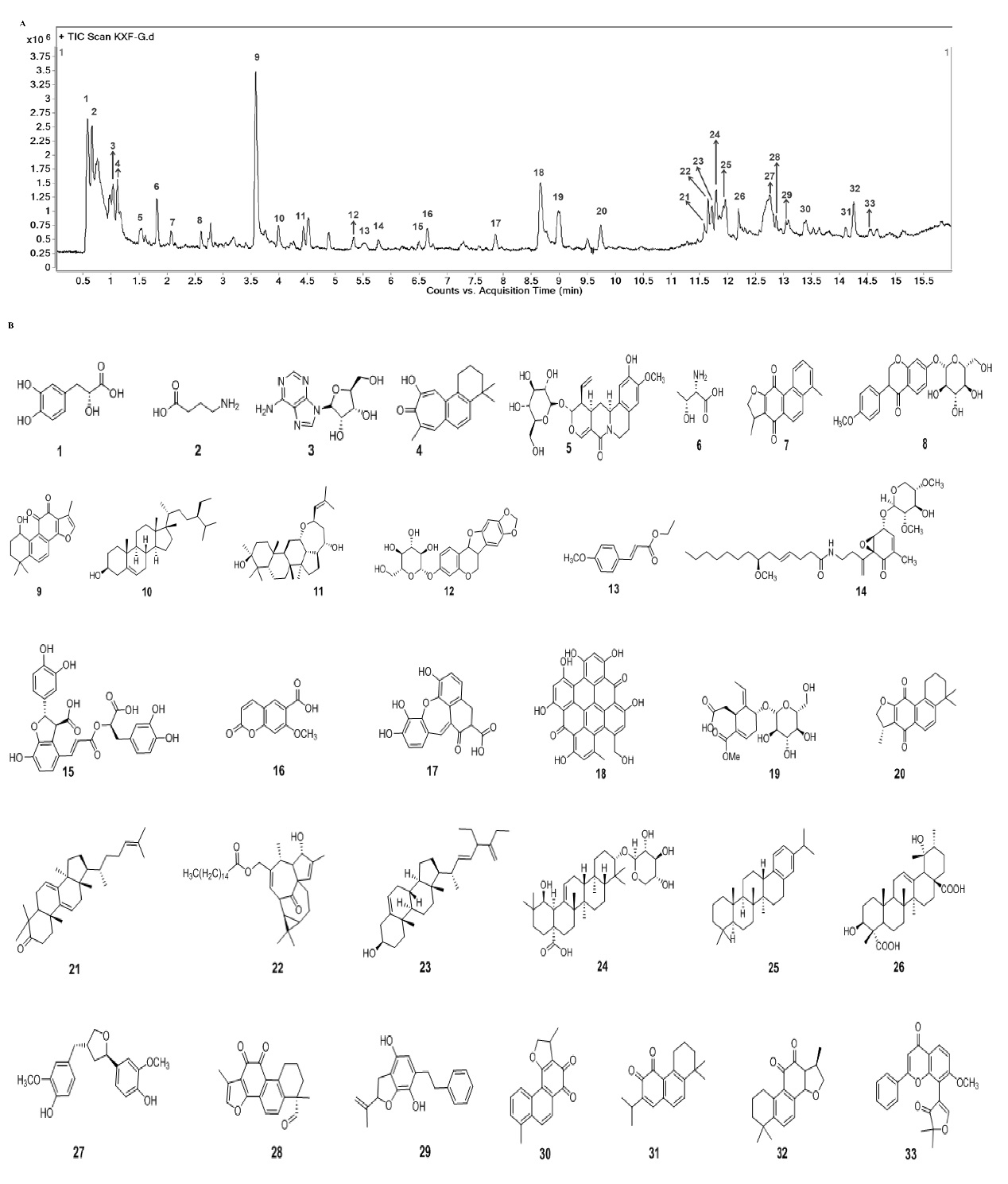


**Figure S1 Chemical composition in QWQX І.** (A)The chromatogram of the QWQX І (B) Assigned structures of 33 characteristic compounds in QWQX І.


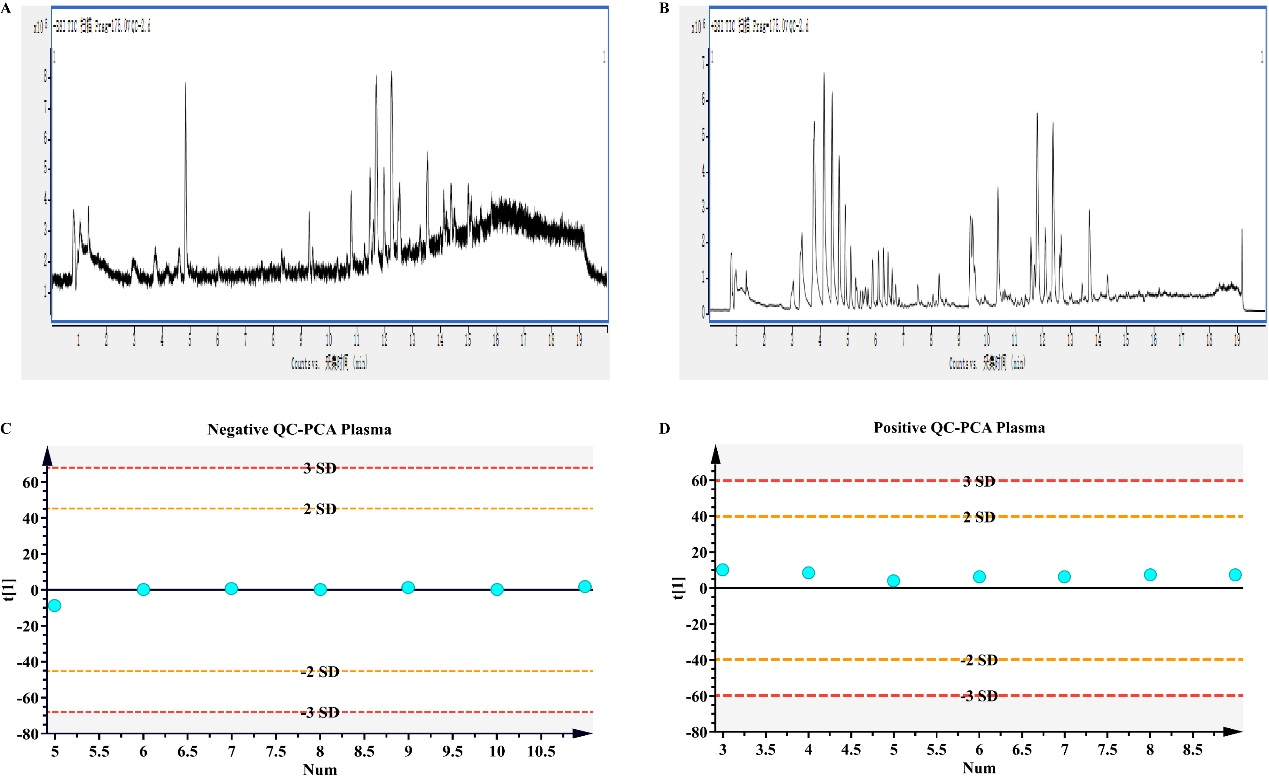


**Figure S2 Metabolic profile of rat plasma.** (A) Total ion flow chromatogram in negative mode; (B) Total ion flow chromatogram in positive mode; (C) Quality control samples PCA scores in negative mode; (D) Quality control samples PCA scores in positive mode.


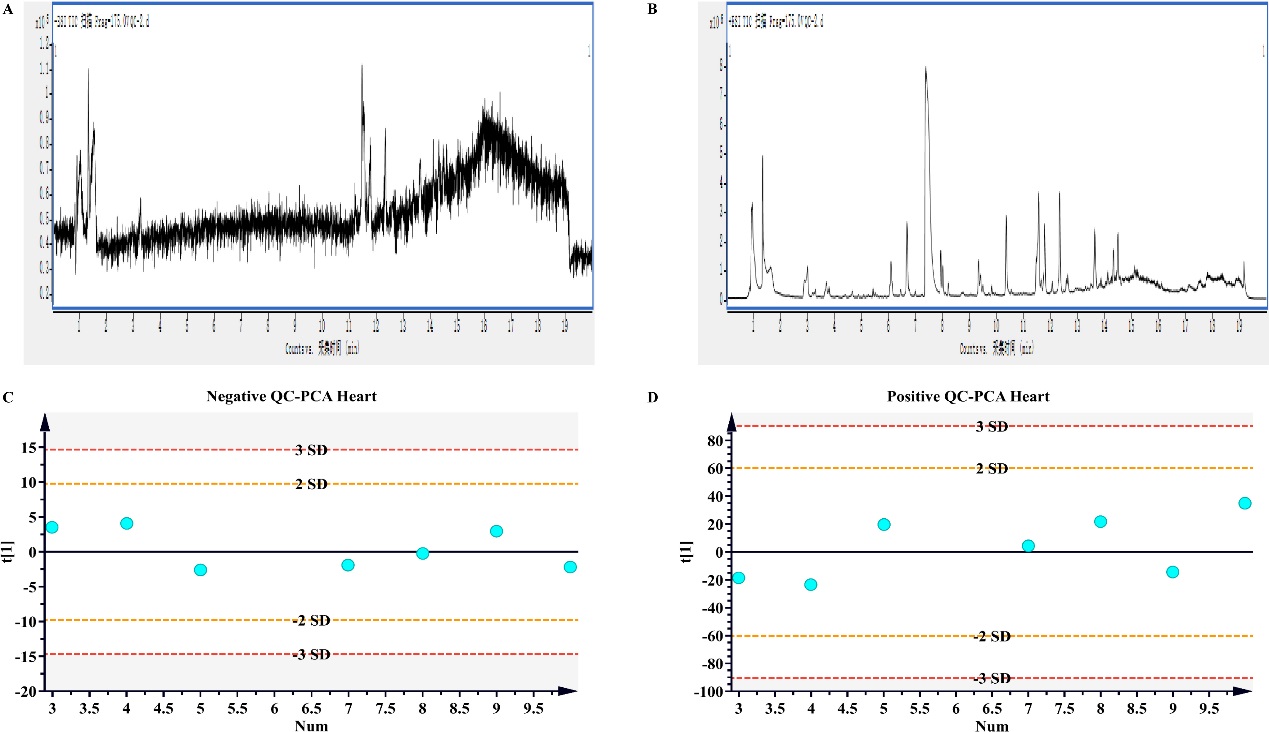


**Figure S3 Metabolic profile of rat cardiac tissue.** (A) Total ion flow chromatogram in negative mode; (B) Total ion flow chromatogram in positive mode; (C) Quality control samples PCA scores in negative mode; (D) Quality control samples PCA scores in positive mode.
