# Supplementary material for: Exploring the mechanism of anti-chronic heart failure effect of qiweiqiangxin І granules based on metabolomics
Source: Front Pharmacol. 2023 Feb 13;14:1111007. doi: 10.3389/fphar.2023.1111007 (PMC9968974; doi:10.3389/fphar.2023.1111007)
Supplement: Supplementary file 2 [file Table2.DOCX]

**Raw Data**

**Exploring the mechanism of anti-chronic heart failure effect of Qiweiqiangxin І granules based on metabolomics**

**Wanru Zhong^1†^, Yihua Li^3†^, Haixiang Zhong^1^, Yuanyuan Cheng^1^, Qi Chen^1,2^, Xinjun Zhao^2^, Zhongqiu Liu^1*^, Rong Li ^2*^, Rong Zhang ^1*^**

^1^Guangdong Provincial Key Laboratory of Translational Cancer Research of Chinese Medicines, Joint International Research Laboratory of Translational Cancer Research of Chinese Medicines, International Institute for Translational Chinese Medicine, School of Pharmaceutical Sciences, Guangzhou University of Chinese Medicine, Guangzhou, China.

^2^Department of Internal Medicine-Cardiovascular, The First Affiliated Hospital of Guangzhou University of Chinese Medicine, Guangzhou, Guangdong, China

^3^The first clinical medical college, Guangzhou University of Chinese Medicine, Guangzhou, China.

**^†^** These authors have contributed equally to this work and share first authorship

In <https://www.jianguoyun.com/>

<https://www.jianguoyun.com/p/DYBVPvcQubabCxjrr-kEIAA>

<https://www.jianguoyun.com/p/DfHv2L8QubabCxj2r-kEIAA>
